# Comparison of retinal degeneration treatment with four types of different mesenchymal stem cells, human induced pluripotent stem cells and RPE cells in a rat retinal degeneration model

**DOI:** 10.1186/s12967-023-04785-1

**Published:** 2023-12-14

**Authors:** Qian Liu, Jun Liu, Minmei Guo, Tzu-Cheng Sung, Ting Wang, Tao Yu, Zeyu Tian, Guoping Fan, Wencan Wu, Akon Higuchi

**Affiliations:** 1 State Key Laboratory of Ophthalmology, Optometry and Visual Science, Eye Hospital, Wenzhou Medical University, No. 270, Xueyuan Road, Wenzhou, 325027 Zhejiang China; 2 Department of Human Genetics, David Geffen School of Medicine, UCLA, Los Angeles, CA 90095 USA; 3 Department of Chemical and Materials Engineering, National Central University, No. 300, Jhongda RD., Jhongli, Taoyuan, 32001 Taiwan; 4 Shanghai Institute for Advanced Immunochemical Studies, ShanghaiTech University, 201210 Shanghai, China

**Keywords:** Retinal degeneration, Stem cell therapy, Retinal pigmented epithelium, Royal College of Surgeons rats, Human pluripotent stem cell, Mesenchymal stem cell, Visual function, Subretinal transplantation, Electroretinography

## Abstract

**Background:**

Retinal degeneration (RD) is a group of disorders on irreversible vision loss. Multiple types of stem cells were used in clinical trials for RD treatment. However, it remains unknown what kinds of stem cells are most effective for the treatment. Therefore, we investigated the subretinal transplantation of several types of stem cells, human adipose-derived stem cells (hADSCs), amniotic fluid stem cells (hAFSCs), bone marrow stem cells (hBMSCs), dental pulp stem cells (hDPSCs), induced pluripotent stem cell (hiPSC), and hiPSC-derived retinal pigment epithelium (RPE) cells for protection effects, paracrine effects and treatment efficiency in an RD disease model rats.

**Methods:**

The generation and characterization of these stem cells and hiPSC-derived RPE cells were performed before transplantation. The stem cells or hiPSC-derived RPE cell suspension labelled with CellTracker Green to detect transplanted cells were delivered into the subretinal space of 3-week-old RCS rats. The control group received subretinal PBS injection or non-injection. A series of detections including fundus photography, optomotor response (OMR) evaluations, light–dark box testing, electroretinography (ERG), and hematoxylin and eosin (HE) staining of retinal sections were conducted after subretinal injection of the cells.

**Results:**

Each stem cell, hiPSC-derived RPE cell or PBS (blank experiment) was successfully transplanted into at least six RCS rats subretinally. Compared with the control rats, RCS rats subjected to subretinal transplantation of any stem cells except hiPSCs showed higher ERG waves (*p* < 0.05) and quantitative OMR (qOMR) index values (hADSCs: 1.166, hAFSCs: 1.249, hBMSCs: 1.098, hDPSCs: 1.238, hiPSCs: 1.208, hiPSC-RPE cells: 1.294, non-injection: 1.03, PBS: 1.06), which indicated better visual function, at 4 weeks post-injection. However, only rats that received hiPSC-derived RPE cells maintained their visual function at 8 weeks post-injection (*p* < 0.05). The outer nuclear layer thickness observed in histological sections after HE staining showed the same pattern as the ERG and qOMR results.

**Conclusions:**

Compared to hiPSC-derived RPE cells, adult and fetal stem cells yielded improvements in visual function for up to 4 weeks post-injection; this outcome was mainly based on the paracrine effects of several types of growth factors secreted by the stem cells. Patients with RD will benefit from the stem cell therapy.

**Supplementary Information:**

The online version contains supplementary material available at 10.1186/s12967-023-04785-1.

## Introduction

The loss of vision affects the quality of daily life and psychological state of patients, which places an enormous burden on not only the family but also the National Health Insurance budget. The retina, which consists of the neural retina and the retinal pigment epithelium (RPE), plays a pivotal role in light perception and signal transduction [1]. Retinal degeneration (RD) is a group of diseases characterized by retinal cell degeneration, such as the cell loss of photoreceptors and/or the RPE in retinitis pigmentosa (RP), age-related macular degeneration (AMD) and Stargardt’s macular dystrophy, which are caused by inherited factors or acquired factors with an increasing incidence and prevalence in recent years [2–7].

In inherited RD, thousands of mutations in hundreds of genes have been found [8]. However, only one gene therapy has been approved by the United States Food and Drug Administration (FDA) for the treatment of patients with *RPE65*-related inherited RD [9]. There is no treatment for other inherited RD patients except for patients with RPE65 mutation. In general, it is difficult to obtain approval for gene therapies for clinical trials and routine clinical treatment. For acquired types of RD, such as AMD, only some patients, such as those with wet AMD, show slower progression of the disease after monthly treatment with anti-vascular endothelium growth factor (VEGF), which is extremely expensive [10–12]. Given that only a very small number of patients with RD can be treated with the expensive or monthly treatment, for most of the remaining RD patients, including those with dry AMD, there are currently very few treatment options. Fortunately, with the emergence and advancements in stem cell research, human induced pluripotent stem cell (hiPSC)-derived RPE cells and/or hiPSC-derived photoreceptor cells seem to be promising treatments for RD patients [13–25], since the RPE cells, which are located adjacent to the photoreceptor cells, play a vital role in maintaining the retinal homeostasis and normal vision.

Stem cell therapy for the treatment of RD has been investigated using several types of adult stem cells, such as bone marrow stem cells (BMSCs) [26, 27], dental pulp stem cells (DPSCs) [28–30], adipose-derived stem cells (ADSCs) [31, 32] and neural stem cells (NSCs) [33], as well as stem cell-derived cells, such as hiPSC-derived RPE cells and hiPSC-derived retinal stem and progenitor cells or retinal epithelium cells [34, 35]. However, there has been no systematic study comparing the effects of different types of adult stem cells in RD treatment using an animal model of RD.

Furthermore, there have been very few studies that compared the treatment effects of human adult stem cells (hBMSCs, hDPSCs and hADSCs) or human fetal stem cells (human amniotic fluid stem cells, hAFSCs) and hiPSC-derived RPE cells in animals with RD (RCS rats) [36–38], although clinical trials of the transplantation of specific stem cells or stem cell-derived cells, such as BMSCs (NCT03772938, NCT03011541, NCT02016508, NCT01920867, NCT01736059, and NCT01518127), ADSCs (NCT02024269), umbilical cord-derived mesenchymal stem cells (UC-MSCs; NCT05147701), human central nervous system stem cells (HuCNS-SCs; NCT02467634, NCT02137915, and NCT01632527), retinal stem and progenitor cells (NCT05187104), and stem cell-derived RPE cells (NCT04627428, NCT04339764, NCT03305029, NCT03178149, NCT03102138, NCT03046407, NCT02941991, NCT02903576, NCT02755428, NCT02749734, NCT02590692, NCT02563782, NCT02464956, NCT02463344, NCT02445612, NCT02286089, NCT02122159, NCT01691261, NCT01674829, NCT01625559, NCT01469832, NCT01345006, and NCT01344993) for the treatment of RD patients have been conducted [39].

Among the many studies that have investigated stem cell therapy using animal models of RD, few studies have compared the protective effects or improvements in efficacy achieved in rats or mice with RD using only two or three different types of stem cells or stem cell-derived cells [33, 40]. Mead et al. compared the abilities of three different types of human adult stem cells (DPSCs, BMSCs and ADSCs) to protect retinal ganglion cells in vitro [40]. However, they did not compare the treatment effects of adult stem cells and hiPSC-derived RPE cells on retinal ganglion cells.

Sun and Takahashi et al. compared the neuroprotective efficacy of three cell types [hiPSC-RPE cells, BMSCs, and neural stem cells (NSCs)] in RD treatment in an immunocompromised mouse model, *rd1* mice [33]. However, they did not evaluate the neuroprotective efficacy of several kinds of adult and/or fetal stem cells, such as ADSCs, DPSCs, AFSCs and BMSCs, but instead investigated this issue using only one cell line, BMSCs.

Xu et al. compared two subpopulations of rat BMSCs in terms of their ability to protect against RD progression in an animal model using Royal College of Surgeons (RCS) rats [36] and later compared the protective effects of two subpopulations of human UC-MSCs [38]. They did not investigate the protective effects of other types of stem cells, such as ADSCs, DPSCs and AFSCs, against RD progression.

Riera et al. compared the treatment effects of transplantation of two different RPE cell lines, which were differentiated from human embryonic stem cells (hESCs) and human induced pluripotent stem cells (hiPSCs), for RD in RCS rats [37]. However, they did not compare the treatment effects of several types of adult and/or fetal stem cells with those of hESCs or hiPSC-derived RPE cells in the context of RD.

To our knowledge, there have been no studies investigating the protective effects and treatment efficacy of subretinal transplantation of several types of stem cells (hAFSCs, hDPSCs, hADSCs, hBMSCs, and hiPSCs) as well as hiPSC-derived RPE cells using an animal model of RD (RCS rats). RCS rats are a well-recognized and classical animal model of RD, which is caused by the mutation of the MER proto-oncogene, tyrosine kinase (*Mertk*); in this model, RPE cells cannot phagocytose the outer segment of photoreceptor cells, which leads to progressive death of photoreceptor cells [41–44]. Apoptosis of photoreceptor cells begins at approximately 21 days after birth in RCS rats, and the photoreceptor cells almost die when the rats are 2 to 3 months of age, which leads to severe loss of retinal structure and function [37, 45, 46].

In this study, we investigated and compared the subretinal transplantation of several types of stem cells (hAFSCs, hDPSCs, hADSCs, hBMSCs, and hiPSCs) as well as hiPSC-derived RPE cells to determine the efficacy and protective effects of stem cells and hiPSC-derived RPE cells and to investigate their mechanisms in the treatment of RD in RCS rats. All the data manifested that stem cell based regenerative medicine, especially hiPSCs-derived RPE cells transplantation, showed excellent functional and structural recovery in RCS rats, which will be a promising treatment for clinical RD patients.

## Methods

### Experimental models and study participant details

#### Rats

RCS rats (3-week age), a well-recognized and classical animal model of RD, were used in this study. RCS rats were maintained on 12-h light–dark cycle. The rats were allowed to drink water, eat food and move freely in transparent rat cages. Animal experiments were approved by the Laboratory Animal Ethics Committee of Wenzhou Medical University (No. wydw2021-0230), and all procedures were based on the guidelines of the Laboratory Animal Center of Wenzhou Medical University.

#### Cell sources and cultivation

The experiments in this study were approved by the ethics committees of Wenzhou Medical University (wydw2022-0474). The preparation of hAFSCs from fresh second-trimester amniotic fluid was performed using our previously reported method [47]. Briefly, the fresh amniotic fluid samples were centrifuged for 5 min at 260 *g* to get the cell pellets. Then the cell pellets were resuspended with primary mesenchymal stem cell culture medium (PriMed-iCell-012, iCellbioscience, Shanghai, China), and the cells were cultured until the passage. hAFCs at passage 4 were used in the following experiments. The preparation of hDPSCs from dental pulp was also performed using our previously reported method [48]. Briefly, the teeth were sterilized using 2% streptomycin-penicillin for 5–10 min. Then, the teeth to expose and get the inner dental pulp. Subsequently, the dental pulp tissue was washed with PBS and suspended in PriMed-iCell-012 medium, and the cells were cultured until the passage. hDPSCs at passage 4 were used in the following experiments.

hBMSCs and hADSCs were purchased from iCellbioscience Company (Shanghai, China). The hiPSC (HPS0077) cell line was obtained from RIKEN BioResource Center (Tsukuba, Japan). hAFSCs, hDPSCs, hBMSCs, and hADSCs were cultured and passaged using PriMed-iCell-012 medium. HPS0077 cells were cultivated and expanded on plates coated with Matrigel (354230, Corning, NY, USA) using mTESR1 medium (85850, Stemcell Technologies, Vancouver, Canada).

### Method details

#### Study design

The purpose of this study was to investigate the effects of different stem cells (hAFSCs, hDPSCs, hADSCs, hBMSCs, and hiPSCs) and hiPSC-derived RPE cells in RD treatment by subretinal transplantation of each cell type in an RD animal model (RCS rats).

**Fig. 1:**
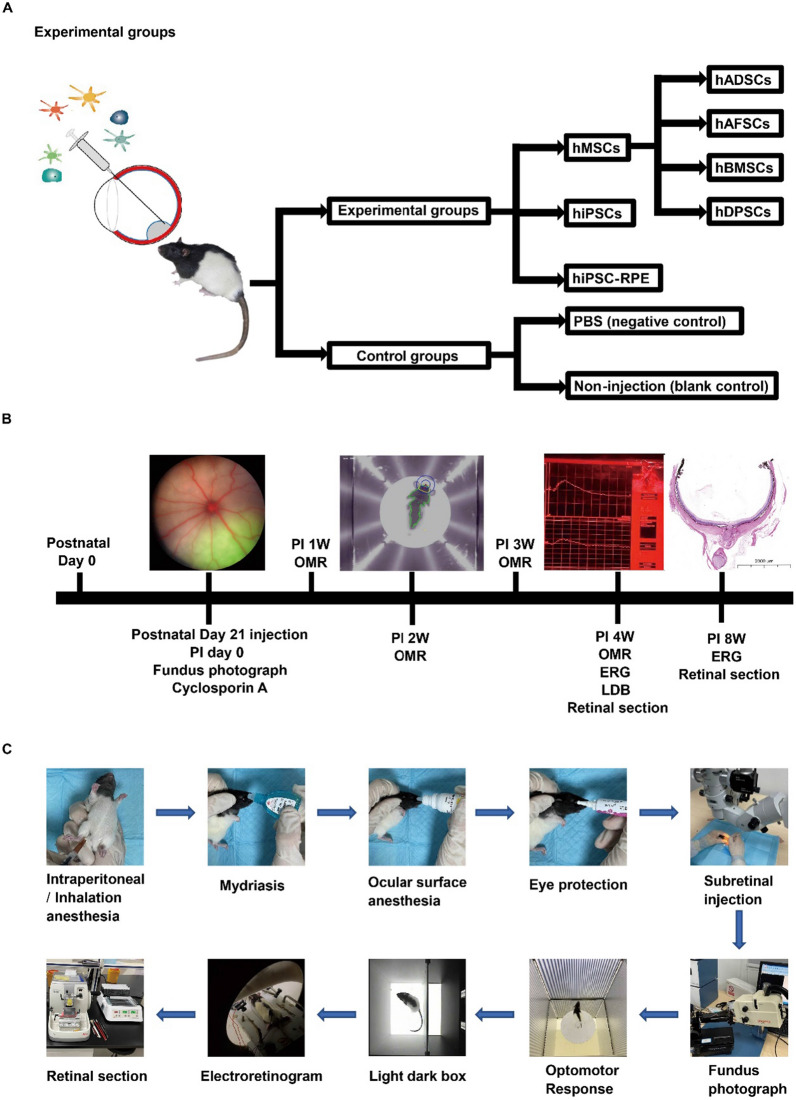
Schematic diagram of the experimental design and the process used to evaluate RCS rats in this study. **A** The experimental design for subretinal injection of different cells into Royal College of Surgeons (RCS) rats, which included four kinds of human mesenchymal stem cells (hMSCs), human induced pluripotent stem cells (hiPSCs), and hiPSC-derived retinal pigment epithelium cells (hiPSC-RPE cells). *hADSCs* human adipose-derived stem cells, *hAFSCs* human amniotic fluid stem cells, *hBMSCs* human bone marrow stem cells, *hDPSCs* human dental pulp stem cells, *PBS* phosphate-buffered saline. **B** The timeline of subretinal injection and detection of cells in RCS rats, visual functions were detected by optomotor response (OMR), light–dark box (LDB), and electroretinogram (ERG). *PI* postinjection. **C** The protocol for subretinal injection and detection of cells in RCS rats

Both male and female littermates of RCS rats at postnatal day 21 were randomly allocated to transplantation groups (subretinal transplantation of human stem cells (at least 3 male and 3 female rats on each stem cell), hAFSCs, hDPSCs, hADSCs, hBMSCs, hiPSCs (HPS0077), or hiPSC (HPS0077)-derived RPE cells), a negative control group (subretinal injection of PBS, at least 3 male and 3 female rats), and a blank control group (age-matched noninjection group, at least 3 male and 3 female rats) (Fig. 1A). There were six RCS rats in each group subjected to cell transplantation (10^5^ cells in each condition). The timeline of this study is presented in Fig. 1B. The preset evaluations of RCS rats included fundus photography, optomotor response (OMR) evaluations, light–dark box (LDB) testing, electroretinography (ERG), and histological tests after subretinal cell transplantation (Fig. 1C).

#### hiPSC differentiation into RPE cells

hiPSCs (HPS0077) were differentiated into RPE cells using protocols developed by Maruotti et al. [49] and Smith et al. [50] with some modifications to reduce the cell toxicity of small molecules during differentiation. Briefly, hiPSCs were cultured on Matrigel-coated dishes in mTESR1 medium until the cells reached confluence. Then, the culture medium was switched to RPE differentiation medium (DM), and this time point was the first day (D1). The DM (500 mL) was composed of 425 mL DMEM/F12 (11330, Thermo Fisher Scientific, Waltham, MA, USA) and 75 mL KnockOut Serum Replacement (KSR) (10828, Thermo Fisher Scientific, Waltham, MA, USA), which were supplemented with 5 mL MEM nonessential amino acids (NEAA, 100 ×) (11140, Thermo Fisher Scientific, Waltham, MA, USA), 5 mL glutamine (100 ×) (25030, Thermo Fisher Scientific, Waltham, MA, USA), 5 mL antibiotic–antimycotic (anti-anti, 100 ×) (15240, Thermo Fisher Scientific, Waltham, MA, USA), and 3.5 μL β-mercaptoethanol (M3148, Sigma‒Aldrich, St. Louis, MO, USA). At D2 to D14, 10 mM nicotinamide (NIC, Sigma) and 10–50 nM chetomin (CTM, C9623, Sigma‒Aldrich, St. Louis, MO, USA), which increased 10 nM from 10 to 50 nM every 3 days, were also added to the DM. After D14, DM supplemented with only 10 mM NIC was used, and the medium was replaced with fresh DM every day for another 2 weeks. On D28, the medium was replaced by RPE medium until mature and pigmented RPE cells were observed. The RPE medium (500 mL) was composed of 350 mL DMEM (11965, Thermo Fisher Scientific, Waltham, MA, USA) and 150 mL F12 (11765, Thermo Fisher Scientific, Waltham, MA, USA), which was supplemented with 10 mL B27 (50 ×) (17504, Thermo Fisher Scientific, Waltham, MA, USA) and 5 mL Anti-Anti (100 ×).

#### Flow cytometry analysis of the cells

The hADSCs, hAFSCs, hBMSCs, and hDPSCs were MSCs, thus those cells were stained with antibodies against MSC markers (FITC-labeled anti-CD44 (11-0441-82, Thermo Fisher Scientific, Waltham, MA, USA), PE-labeled anti-CD73 (12-0739-42, Thermo Fisher Scientific, Waltham, MA, USA), and PE-labeled anti-CD105 (12-1057-42, Thermo Fisher Scientific, Waltham, MA, USA), antibodies against a human hematopoietic progenitor marker (negative control) [FITC-labeled anti-CD34 (11-0349-41, Thermo Fisher Scientific, Waltham, MA, USA), FITC-labeled isotype (11-471482, Thermo Fisher Scientific, Waltham, MA, USA), and PE-labeled isotype (12-4714-82, Thermo Fisher Scientific, Waltham, MA, USA)] at the recommended concentrations using 5 µL (0.5 µg)/test, following a previously described method [48] for flow cytometry analysis of MSC markers on cells.

The hiPSC-derived RPE cells were dissociated into single cells using 0.05% trypsin–EDTA phenol red (25200, Gibco, Grand Island, NY, USA) for 30 min at 37 °C. Then, the cells were treated with 4% paraformaldehyde (PFA) for 20 min and subsequently immersed in 90% methanol for 15 min at room temperature (RT). The cells were stained with the following RPE-related primary antibodies: anti-MiTF (ab3201, mouse, Abcam, Cambridge, UK), anti-PAX6 (MA532409, rabbit, Thermo Fisher Scientific, Waltham, MA, USA), anti-RPE65 (MA116578, mouse, Thermo Fisher Scientific, Waltham, MA, USA mouse), anti-ZO1 (402200, rabbit, Thermo Fisher Scientific, Waltham, MA, USA), mouse isotype antibodies (ab81216, mouse, Abcam, Cambridge, UK), and rabbit isotype antibodies (ab172730, rabbit, Abcam, Cambridge, UK) at a concentration of 1 μg/10^6^ cells for 1 h at RT. Subsequently, the cells were stained with secondary antibodies (anti-mouse or anti-rabbit) conjugated with PE (1:500) or FITC (1:500) according to the species (mouse or rabbit) of the primary antibody [anti-mouse IgG H&L (FITC, ab6785, goat, Abcam, Cambridge, UK) and anti-rabbit IgG H&L (PE, ab72465, goat, Abcam, Cambridge, UK)]. Before starting each step, the cells were washed three times with Dulbecco's phosphate-buffered saline (DPBS). A BD C6 plus flow cytometer (BD Biosciences, USA) was used for the detection and analysis of cells stained with antibodies.

#### Immunofluorescence analysis of the cells

The hiPSCs and hiPSC-derived RPE cells were passaged on Matrigel-coated 3.5 cm cell culture dishes with glass bottoms for confocal laser scanning microscopy (LSM880 with Airyscan, ZEISS, Germany) observation. The cells were fixed with 4% PFA for 30 min at RT, followed by permeabilization with 0.1% Triton X-100 (P0096, Beyotime, Shanghai, China) for 15 min and treatment with blocking buffer (P0260, Beyotime, Shanghai, China) for 30 min at RT. Subsequently, hiPSCs were incubated with primary antibodies (1:200) specific for pluripotent markers at 4 °C overnight: anti-Nanog antibodies (MA1017, mouse, Thermo Fisher Scientific, Waltham, MA, USA), anti-OCT4 antibodies (PA527438, rabbit, Thermo Fisher Scientific, Waltham, MA, USA), and anti-SSEA4 antibodies (MA1021, mouse, Thermo Fisher Scientific, Waltham, MA, USA).

The hiPSC-derived RPE cells were incubated with anti-MiTF antibodies (ab3201, mouse, Abcam, Cambridge, UK), anti-PAX6 antibodies (MA532409, rabbit, Thermo Fisher Scientific, Waltham, MA, USA), anti-RPE65 antibodies (MA116578, mouse, Thermo Fisher Scientific, Waltham, MA, USA), and anti-ZO1 antibodies (402200, rabbit, Thermo Fisher Scientific, Waltham, MA, USA) at 4 °C overnight. On day 2, the cells were incubated with the secondary antibody (1:1000), goat anti-rabbit IgG H&L (Alexa Fluor® 488, ab150077, Abcam, Cambridge, UK) or goat anti-mouse IgG H&L (Alexa Fluor® 594, ab150116, Abcam, Cambridge, UK), for 2 h at RT in the dark, followed by staining with 4′,6-diamidino-2-phenylindole (DAPI) for 5 min. Before each step, the cells were washed 3 times with DPBS. Immunofluorescence images were taken with a confocal laser scanning microscope, using the following settings: three different ranges of laser wavelength, including excitation wavelength 561 nm (channel 1, red), 488 nm (channel 2, green), and 405 nm (channel 3, blue) were set for three different channels.

#### Enzyme-linked immunosorbent assay (ELISA) for trophic factors secreted by cells

The culture medium was collected from confluent cell samples, including hAFSCs, hDPSCs, hADSCs, hBMSCs, HPS0077, and hiPSC-derived RPE cells, at 48 h after the medium change. Then, the medium was centrifuged at 1000 ×*g* for 20 min to obtain debris-free supernatant and stored at − 20 °C or – 80 °C until use. In addition, cell numbers were counted and recorded for subsequent calculations. The secreted levels of human Brain-Derived Neurotrophic Factor (BDNF), human Glial Cell Line-Derived Neurotrophic Factor (GDNF), human pigment epithelium derived factor (PEDF), human Vascular Endothelial Growth Factor A (VEGFA), human Transforming Growth Factor-β (TGF-β), human Hepatocyte Growth Factor (HGF), and human Fibroblast Growth Factor 2 (FGF2, bFGF) were evaluated with a human BDNF-ELISA kit (JL11683, Jianglaibio, Shanghai, China), GDNF-ELISA kit (JL12988, Jianglaibio, Shanghai, China), PEDF-ELISA kit (JL10799, Jianglaibio, Shanghai, China), VEGF-ELISA kit (JL18341, Jianglaibio, Shanghai, China), TGF-β-ELISA kit (JL20082, Jianglaibio, Shanghai, China), HGF-ELISA kit (JL10756, Jianglaibio, Shanghai, China), and FGF2-ELISA kit (JL14546, Jianglaibio, Shanghai, China), respectively, according to the manufacturer’s instructions. Standard protein solution having gradient concentration in the kit were used to generate standard curve for calculating the concentration of trophic factors. The secretions of trophic factors of different cells were normalized by deducting the corresponded control (cell culture medium only). A total of three independent samples were collected from each cell line for testing and statistical analysis.

#### Subretinal transplantation of cells into RCS rats

Stem cells or hiPSC-derived RPE cells were transduced with lentivirus-green fluorescent protein (GFP) or incubated with 20 μM CellTracker Green probe (C2925, Thermo Fisher Scientific, Waltham, MA, USA) in a cell incubator for 30 min. Subsequently, each cell type (hAFSCs, hDPSCs, hADSCs, hBMSCs, hiPSCs, and hiPSC-derived RPE cells) was counted, washed, and resuspended at a final concentration of 5 × 10^4^/μL in DPBS.

RCS rats at postnatal day 21 were chosen for subretinal transplantation. All subretinal transplantations were performed by the same surgeon in this study. The littermates of RCS rats were randomly divided into groups, with six RCS rats in each group. Either abdominal anesthesia with 1% pentobarbital sodium (30 mg/kg) or sustaining inhalational anesthesia with isoflurane (2% at 4 L/min fresh gas flow) was used depending on the experiment. Compound tropicamide eye drops (Qiukang, Handan Kangye Pharmaceutical) were used to dilate the pupils, followed by proparacaine hydrochloride eye drops (Alcaine, Alcon) for ocular surface anesthesia and Ofloxacin eye ointment (Dikeluo, Sinqi Pharmaceutical) to prevent dry eye and bacterial infection. Subsequently, the rats were placed under a surgical microscope (M620F20, Leica Microsystems, Wetzlar, Germany) for cell transplantation. The first channel was created 1–2 mm outside the limbus by using a 29G insulin needle. Then, 2 μL (containing a total of 10^5^ cells labeled with CellTracker Green probe or transduced with lentivirus-GFP) of cell suspension was smoothly and evenly transplanted into the subretinal space of the right eye with a 33G Hamilton blunt needle and a 5-μL Hamilton syringe (Fig. 1C). For the negative control group, the rats received 2 μL DPBS (containing the same concentration of CellTracker Green probe) by subretinal injection into the right eye. Other untreated rats were used as blank controls. After surgery, the rats were placed on a thermostat plate at 37 °C until they were revived. The immunosuppressive drug cyclosporine A (C106893, Aladdin, Shanghai, China) was added to the drinking water of all groups of rats at a concentration of 210 mg/L after transplantation until the rats were sacrificed for the evaluation of eyeball sections.

#### Fundus photography to evaluate RCS rats

Fundus photographs of the eyes of each RCS rat were taken using a Micron IV retinal imaging microscope (Phoenix Research Labs, Pleasonton, CA) to determine whether the transplantation of the cells was successful immediately after transplantation of the cells into RCS rats. Bright field images and fluorescence images were captured in channel 1 (bright) and channel 2 (green fluorescence), respectively. In the transplantation groups, rats with green fluorescent blebs in the subretinal space were defined as successfully injected. In the PBS injection group, rats with subretinal blebs were detected.

#### Optomotor response evaluation in RCS rats

The quantitative optomotor response (qOMR) was recorded for RCS rats subjected to subretinal transplantation of each type of stem cell or hiPSC-derived RPE cells at 1, 2, 3 and 4 weeks post-injection. Rats were placed on the white platform of the qOMR system (PhenoSys, Berlin, Germany) with four screens on each wall in the square box (Fig. 1C). The stimulation protocols were as follows: the spatial frequency of the pattern was set at full check with several different spatial frequencies (0.05, 0.1, 0.15, 0.2, 0.25, 0.3, 0.35, 0.375, 0.4, 0.425, 0.45, and 0.5 cycles/degree), with movement at 12 degree/s for 60 s. Subsequently, the head movements of the rats following movement patterns were automatically tracked and calculated on the qOMR system, and the correct/incorrect tracking behaviors were defined as the qOMR index.

#### Light–dark box evaluation of RCS rats

Behavioral evaluation of RCS rats was performed in a platform called a light–dark box in natural light at 4 weeks post-transplantation of different cells. Two distinct arenas in this platform were defined as the dark chamber and the light chamber using an EthoVision® XT system (Noldus Information Technology, Wageningen, Netherlands). The rats were allowed to move freely between the chambers by using the gate between the two chambers. The light–dark box was connected to a CCD camera above the chamber, which was used to capture and monitor the movements of the rats (Fig. 1C) [51, 52]. The infrared light at the bottom of the chamber improved the tracking accuracy. The movements of each rat were detected for 5 min. The duration of time spent in each chamber was calculated, and a heatmap of the movement track was generated using EthoVision® XT.

#### Electroretinography evaluation of RCS rats

The electrical response of light-sensitive cells in RCS rats transplanted with the investigated cells was recorded by electroretinography (ERG) (RETI-Port21, Roland, Germany) at week 4 and week 8 post-transplantation or at the time when the age of the control RCS rats was matched to that of the rats in the transplantation group to evaluate the visual function of the rats. The rats were placed in a dark room for dark adaptation overnight before the electroretinography test (Fig. 1B and C). The rats were anesthetized by intraperitoneal injection of 1% pentobarbital sodium (30 mg/kg) on day 2. The rats were placed on a thermostat platform at 37 °C to keep them warm throughout the ERG test. The pupils of the rats were dilated fully with compound tropicamide eye drops every 5 min for at least 3 applications, followed by treatment with Alcaine eye drops for ocular surface anesthesia and ofloxacin eye ointment to prevent dry eyes and bacterial infection of the eyes. Two gold loop electrodes (positive electrodes) were placed on the binocular cornea. Two needle reference electrodes (negative electrodes) were inserted under the skin of both cheeks. One ground electrode was inserted under the skin of the tail. Then, dark/scotopic-adapted ERGs were recorded under the following stimulus light intensities: 0.01, 3.0, and 10.0 cd/m^2^ in sequence [53]. After the tests, the rats were placed on a 37 °C thermostat plate until they were revived. All procedures for the dark/scotopic-adapted ERG tests were performed in dim red light.

#### Sections of paraffin-embedded rat eyeballs stained with hematoxylin–eosin (HE)

The rats were sacrificed after the ERG test, and the eyeballs of the rats were enucleated at 4 and 8 weeks after cell transplantation. The eyeballs of the rats were fixed using Davidson’s fixative (PH0975, Phygene, Fuzhou, China) protocol at 4 °C overnight, as reported previously [18, 54]. The cornea and iris were removed before dehydration. After gradient dehydration using graded ethanol in a tissue dehydrator (HistoCore PEARL, Leica Microsystems, Wetzlar, Germany), the lenses were removed, and the eyes were immersed in paraffin. Subsequently, the retinas embedded in paraffin were sliced into 5-micron retinal sections using a paraffin slicing machine (BIOCUT, Leica Microsystems, Wetzlar, Germany). The sliced sections were immersed in ultrapure water at RT and then flattened at 55 °C for 10 s. Adhesion microscope slides (188105, Citoglas, Jiangsu, China) were used to load the sliced sections, and then the slides were placed on a slide drier for 6 h. Only retinal sections that crossed the optic nerve head (ONH) were retained for hematoxylin–eosin (HE) staining and subsequently used for observation. HE staining was conducted following the conventional procedure using an autostainer (Autostainer XL, ST5010, Leica Microsystems, Wetzlar, Germany). After HE staining of the samples, the slides were sealed using neutral balsam and a cover slip. Images were taken using a Pannoramic SCAN II digital microscope (3DHISTECH, Budapest, Hungary).

#### Histological analysis of the retina

The retinal thickness of the outer nuclear layer (ONL) and the number of nuclei per column in the ONL were determined in this study. A total of 14 positions, with each point at a 200 μm interval from the optic nerve head (ONH), were selected for measurement for each section. Both the measurement at each point and the average measurement of all 14 points were evaluated and analyzed.

#### Quantification and statistical analysis

All the data are presented as the mean ± standard deviation (SD). All analysis were performed in a masked fashion to reduce bias. Multiple t tests were used for statistical analysis based on the size of the sample investigated in this study. *p < 0.05 was considered to indicate a significant difference, whereas “ns” was used to indicate a difference that was not statistically significant. GraphPad Prism 8.0 (GraphPad Software Inc., La Jolla, CA) and Adobe Illustrator were used for graphing.

## Results

### Characterization of hMSCs, hiPSCs, and hiPSC-RPE cells in vitro

We performed subretinal transplantation of several types of stem cells (hAFSCs, hDPSCs, hADSCs, hBMSCs, and hiPSCs) and hiPSC-derived RPE cells into a rat model of RD disease (RCS rats) and evaluated the efficacy of each stem cell type compared to that of hiPSC-derived RPE cells. Before transplantation of each type of stem cell investigated in this study, the stem cells were characterized and evaluated by flow cytometry and immunofluorescence assays.

**Fig. 2:**
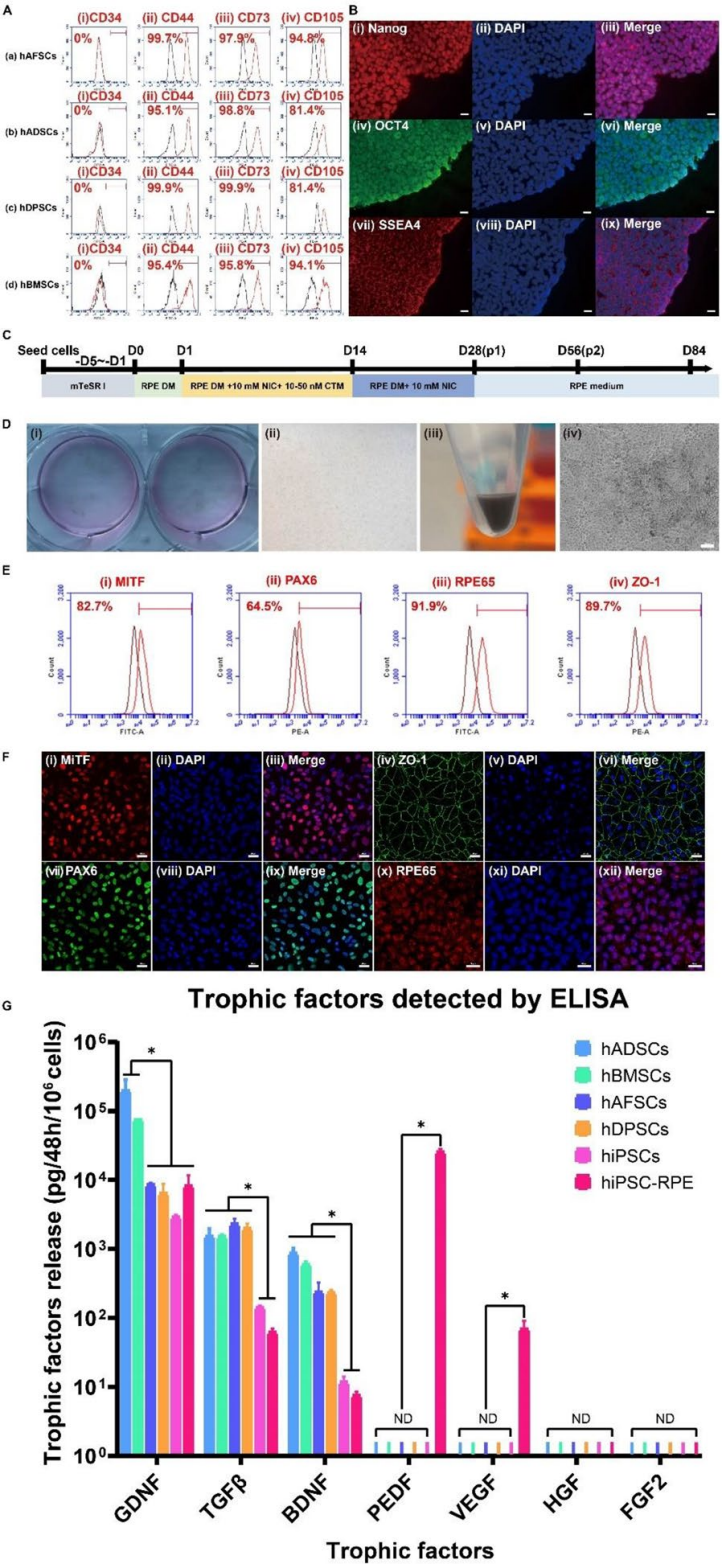
Characterization of hMSCs, hiPSCs, and hiPSC-RPE cells in vitro. **A** Expression of CD34 (a human hematopoietic progenitor marker, negative control) (i), CD44 (a hMSC marker) (ii), CD73 (a hMSC marker) (iii), and CD105 (a hMSC marker) (iv) on (a) hAFSCs, (b) hADSCs, (c) hDPSCs, and (d) hBMSCs using flow cytometry. **B** The expression of the human pluripotent stem cell markers Nanog (i), OCT4 (iv), and SSEA4 (vii) on hiPSCs with nuclear staining of DAPI (ii, v, viii), detected using immunofluorescence. (iii), (vi), and (ix) were generated by merging (I, ii), (iv, v) and (vii, viii), respectively. Scale bar: 20 μm. **C** The timeline of differentiation of hiPSCs into RPE cells. D0: Day 0; *DM* differentiation medium, *NIC* nicotinamide, *CTM* chetomin. **D** The differentiation of hiPSCs into RPE cells. Pigmented cells were observed by the naked eye on day 45 (i) and day 56 (ii). Pigmented cell pellets were observed on day 56 (iii). The morphology of hiPSC-RPE cells (iv). Scale bar: 50 μm. **E** The highly expression of the RPE-related markers MITF (i), PAX6 (ii), RPE65 (iii), and ZO-1 (iv) in hiPSC-RPE cells was analyzed using flow cytometry. **F** Expression of the RPE-related markers MITF (i), ZO-1 (iv), PAX6 (vii), and RPE65 (x) on hiPSC-RPE cells with nuclear staining of DAPI (ii, v, viii, xi), analyzed by immunofluorescence. (iii), (vi), (ix), and (xii) were generated by merging (i, ii), (iv, v), (vii, viii), and (x, xi), respectively. Scale bar: 20 μm. **G** Secretion of the trophic factors GDNF, TGF-β, BDNF, PEDF, VEGF, HGF, and FGF2 by hADSCs, hBMSCs, hAFSCs, hDPSCs, hiPSCs, and hiPSC-RPE cells, expressed as the amount secreted per million cells after two days of culture, as detected by enzyme-linked immunosorbent assay (ELISA). *GDNF* glial cell line-derived neurotrophic factor, *TGFβ* transforming growth factor-β, *BDNF* brain-derived neurotrophic factor, *PEDF* pigment epithelium-derived factor, *VEGF* vascular endothelial growth factor, *HGF* hepatocyte growth factor, *FGF2* fibroblast growth factor 2, *ND* not detected. *p < 0.05

To confirm hAFSCs, hADSCs, hDPSCs, and hBMSCs were successfully derived from different original tissues, we assessed the expression of the three classical mesenchymal stem cell (MSC) surface markers CD44, CD73, and CD105 on human MSCs (hAFSCs, hADSCs, hDPSCs, and hBMSCs) by using flow cytometry. Additionally, the human hematopoietic progenitor marker CD34 was selected as a negative control. The results are shown in Fig. 2A. All of the human MSCs (hMSCs), hAFSCs, hADSCs, hDPSCs, and hBMSCs, showed high expression of MSC surface markers, with over 95, 95, and 90% of the cells positive for CD44, CD73 and CD90 expression, respectively (Fig. 2A). No expression of CD34 (the negative control marker) was detected on hAFSCs, hADSCs, hDPSCs, or hBMSCs (Fig. 2A). Therefore, these four cell types were qualified as stem cell (hMSC) candidates for subsequent cell transplantation experiments.

For hiPSCs (HPS0077), we evaluated the expression of the human pluripotent markers Nanog, OCT4, and SSEA4 by using an immunofluorescence assay. Expression of Nanog, OCT4, and SSEA4 was clearly observed (Fig. 2B), which indicated the pluripotency of hiPSCs.

We generated hiPSC-derived RPE (hiPSC-RPE) cells from hiPSCs (HPS0077) by using a previously reported protocol with some modifications [49, 50] (Fig. 2C). Pigmented hiPSC-RPE cells began to appear around day 45 of differentiation and were abundant on day 56, as observed by the naked eye (Fig. 2Di, ii). hiPSC-RPE cells were passaged on day 56 using 0.05% trypsin–EDTA and subsequently expanded. Cell pellets of hiPSC-RPE cells on day 56 also clearly showed the presence of black pigmented deposits (Fig. 2Diii). Furthermore, the polygonal morphology of RPE cells was observed among hiPSC-RPE cells under a microscope (Fig. 2Div). Moreover, hiPSC-RPE cells expressed RPE-related markers, such as MiTF, PAX6, ZO-1, and RPE65, which were detected by both flow cytometry (Fig. 2E) and immunofluorescence (Fig. 2F) on day 84 of differentiation. All the characterizations of hiPSC-RPE cells confirmed the successful differentiation of hiPSCs into mature RPE cells with the expression of RPE-specific markers, which laid the foundation for the following subretinal transplantation treatments in RCS rats.

The secretion of several growth factors by each cell type (hAFSCs, hADSCs, hDPSCs, hBMSCs, hiPSCs, and hiPSC-RPE) was also evaluated. The culture medium of each cell type was collected from confluent cell samples at 48 h after the culture medium was changed to detect the secretion of several growth factors using ELISA. All six cell lines secreted human GDNF, TGF-β, and BDNF, but they did so to different degrees (Fig. 2G). hADSCs (200,224.4 ± 85,110.15 pg/mL/10^6^ cells/48 h) and hBMSCs (75,596.72 ± 978.74 pg/mL/10^6^ cells/48 h) secreted larger amounts of GDNF than did hAFSCs (8601.73 ± 519.95 pg/mL/10^6^ cells/48 h), hDPSCs (6457.19 ± 2308.04 pg/mL/10^6^ cells/48 h), hiPSC-RPE cells (8286.74 ± 3387.66 pg/mL/10^6^ cells/48 h), and hiPSCs (2942.56 ± 192.67 pg/mL/10^6^ cells/48 h) (*p* < 0.05).

Four types hMSCs, namely, hADSCs (1547.51 ± 422.46 pg/mL/10^6^ cells/48 h), hBMSCs (1531.64 ± 104.51 pg/mL/10^6^ cells/48 h), hAFSCs (2323.67 ± 434.46 pg/mL/10^6^ cells/48 h), and hDPSCs (2003.37 ± 326.79 pg/mL/10^6^ cells/48 h), released larger amounts of TGF-β than did hiPSC-RPE cells (62.58 ± 7.79 pg/mL/10^6^ cells/48 h) and HPS0077 cells (143.49 ± 8.51 pg/mL/10^6^ cells/48 h) (*p* < 0.05) (Fig. 2G).

Similarly, the secretion of BDNF was higher in the four hMSC groups (BDNF secretion by hADSCs: 876.48 ± 146.21 pg/mL/10^6^ cells/48 h, hBMSCs: 603.58 ± 55.74 pg/mL/10^6^ cells/48 h, hAFSCs: 240.61 ± 86.49 pg/mL/10^6^ cells/48 h, and hDPSCs: 234.31 ± 21.91 pg/mL/10^6^ cells/48 h) than in the hiPSCs (11.84 ± 2.22 pg/mL/10^6^ cells/48 h) and hiPSC-RPE cells (7.59 ± 0.98 pg/mL/10^6^ cells/48 h) (*p* < 0.05) (Fig. 2G).

In addition, hiPSC-RPE cells secreted large amounts of both PEDF (25673.52 ± 2643.69 pg/mL/10^6^ cells/48 h) and VEGF (69.78 ± 20.94 pg/mL/10^6^ cells/48 h), which were not detected in the supernatants of other cells (hMSCs and hiPSCs) within the experimental error (Fig. 2G). The growth factors HGF and FGF-2 were not detected in the supernatants of any cell lines investigated in this study within the experimental error (Fig. 2G).

### Subretinal transplantation of different cell types labeled with CellTracker

**Fig. 3:**
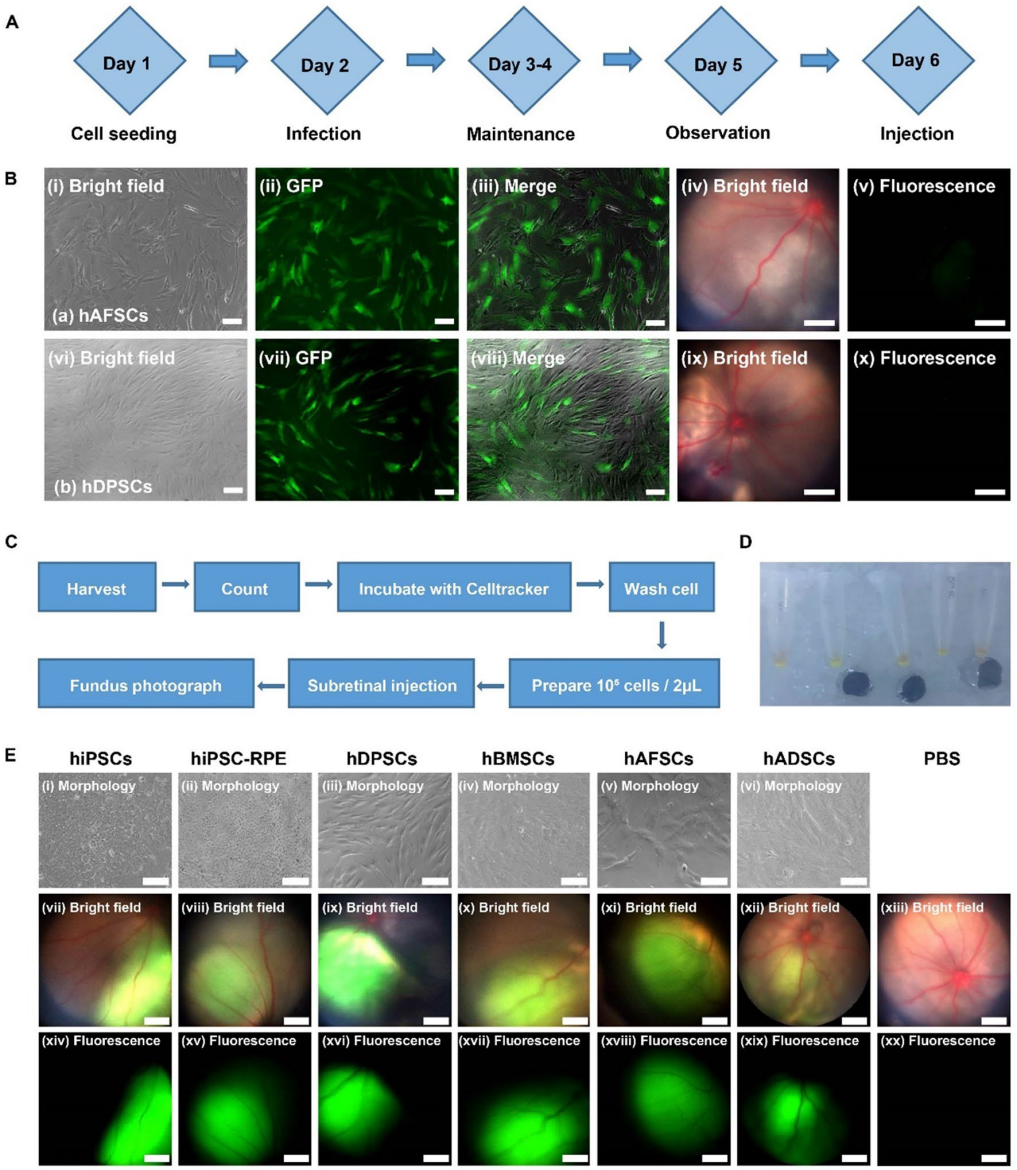
Subretinal transplantation of hMSCs, hiPSCs, and hiPSC-RPE cells. **A** The procedure for cell labeling with lentivirus-GFP. **B** The cell morphology (i, vi) and green fluorescent protein (GFP) expression of hAFSCs (i–iii) and hDPSCs (vi–viii) after lentivirus-GFP transduction. (iii) and (viii) were generated by merging (i, ii) and (vi, vii), respectively. Scale bar: 100 μm. Bright field (iv, ix) and fluorescent channel images (v, x) of fundus photographs of the eyes of RCS rats after subretinal injection of lentivirus-GFP-labeled hAFSCs (iv, v) and hDPSCs (ix, x), where the green fluorescence was hard to find on the fundus photograph of rats subjected to lentivirus-GFP labeled cells. Scale bar: 600 μm. **C** The procedure for cell labeling with CellTracker. **D** Cell pellets labeled with CellTracker on ice. **E** The cell morphology of different cells (i–vi) and fundus photographs (vii-xx) of subretinal injection of different cells and PBS into RCS rats, analyzed in bright field (vii-xiii) and fluorescent channels (xiv–xx) using hiPSCs (i, vii, xiv), hiPSC-RPE cells (ii, viii, xv), hDPSCs (iii, ix, xvi), hBMSCs (iv, x, xvii), hAFSCs (v, xi, xviii), hADSCs (vi, xii, xix), and (g) PBS (xiii, xx), where the fundus photographs of successful cell transplantation were characterized by the obvious green fluorescence within the well-defined retinal eminence, whereas the fundus photograph of control group subjected to PBS showed no fluorescence

Before transplantation of stem cells in this study, lentivirus-GFP was transduced into several types of stem cells, including hAFSCs and hDPSCs, following the manufacturer’s instructions (Fig. 3A). The green fluorescence associated with GFP expression was detected on both hAFSCs and hDPSCs using a microscope in vitro (Fig. 3B). However, green fluorescence was rarely detected in fundus photographs of RCS rats after subretinal transplantation of hAFSCs or hDPSCs (10^5^ cells/2 μL) after transduction of GFP, although substantial retinal eminence and cell masses were observed in fundus photographs (Fig. 3B).

We used the commercially available dye CellTracker green to label different cells on the day of cell transplantation and subsequently detect the cells in vivo (Fig. 3C). Cells labeled with CellTracker green were stored on ice away from light (Fig. 3D), and the cells were transplanted into the subretinal space of RCS rats within 6 h after staining with CellTracker green (10^5^ cells/2 μL) (Additional file 1: Fig. S1.). Subsequently, the eyes of RCS rats were evaluated using fundus photographs. Substantial green fluorescence and retinal eminence were observed in fundus photographs of RCS rats after successful subretinal transplantation of cells such as hiPSCs, hiPSC-RPE cells, hDPSCs, hBMSCs, hAFSCs, and hADSCs (Fig. 3E). Specifically, the fundus photographs of successful cell transplantation were characterized by the well-defined green fluorescence under the retinal vessels and within the subretinal space. The position of the green fluorescence varied with the exact site of transplantation in each rat, and lied on the lower fundus photograph in almost all groups. In addition, a cell mass was observed at the subretinal space of RCS rats subjected to cell injection (Additional file 1: Fig. S1). Rats with Vitreous cavity fluorescence or bleeding were excluded from further study (Additional file 1: Fig. S2.). No fluorescence was detected in the control group after subretinal injection of 2 μL PBS, which is the same volume used to transplant cells stained with CellTracker. No tumor formation was found in the transplanted eyes of each group within the detection period.

### Visual function of RCS rats determined by light–dark box and qOMR assays

**Fig. 4:**
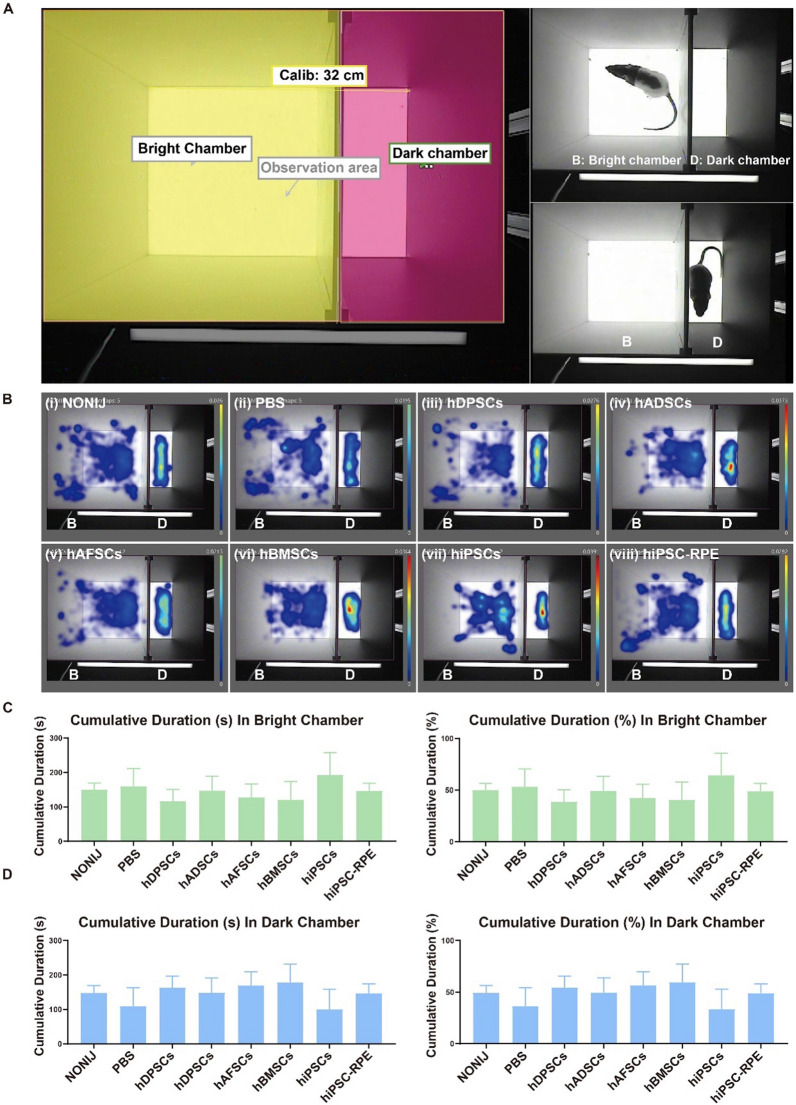
Visual function of RCS rats detected by light–dark box (LDB) testing of groups subjected to subretinal injection of different cells. **A** Schematic of the bright chamber and dark chamber in the LDB in which the RCS rats were placed under natural light condition. **B** Heatmap visualization of the locations of the RCS rats in different groups: non-cell injection (NONIJ, age-matched control) (i), PBS injection (ii), and cell injection of hDPSCs (iii), hADSCs (iv), hAFSCs (v), hBMSCs (vi), hiPSCs (vii), and hiPSC-RPE cells (viii), which revealed a higher frequency of the position of rats by red color, and a lower frequency by more of a blue color. **C** Duration (left figure) and percentage of time (right figure) for which the RCS rats were located in the bright chamber, showing rats that were subjected to subretinal transplantation and rats that were not injected with cells or PBS. **D** Duration for which the RCS rats were located in the dark chamber, showing rats that were subjected to subretinal transplantation and rats that were not injected with cells or PBS

The light and dark box (LDB) assay with two different chambers (a light irradiating chamber and a dark chamber) was used to evaluate the behavior of RCS rats in response to natural light at 4 weeks after transplantation of several types of stem cells and hiPSC-RPE cells subretinally (Fig. 4A) (Additional file 2: Video S1). The locations in which rats were recorded at a higher frequency are shown in red (Fig. 4B). In contrast, the position recorded at a lower frequency is shown as more of a blue color (Fig. 4B). These results demonstrated that the position recorded at the highest frequency for rats, with an index color closer to red or yellow in the heatmap visualization, was located in the dark chamber in all cell transplantation groups and control groups (Fig. 4B). Accordingly, the duration of time spent in the light chamber (Fig. 4C) and dark chamber (Fig. 4D) was not significantly different among all groups (*p* > 0.05), although the red or yellow index color can be seen in the dark chamber region for the cell transplanted groups on the heatmaps, whereas the red or yellow index color could not be detected in the dark chamber for the control groups on the heatmaps. We did not observe a significant difference in the visual behaviors of RCS rats subjected to transplantation of several types of stem cells and hiPSC-RPE cells or no cells at 4 weeks post-transplantation in the light–dark box assay.

**Fig. 5:**
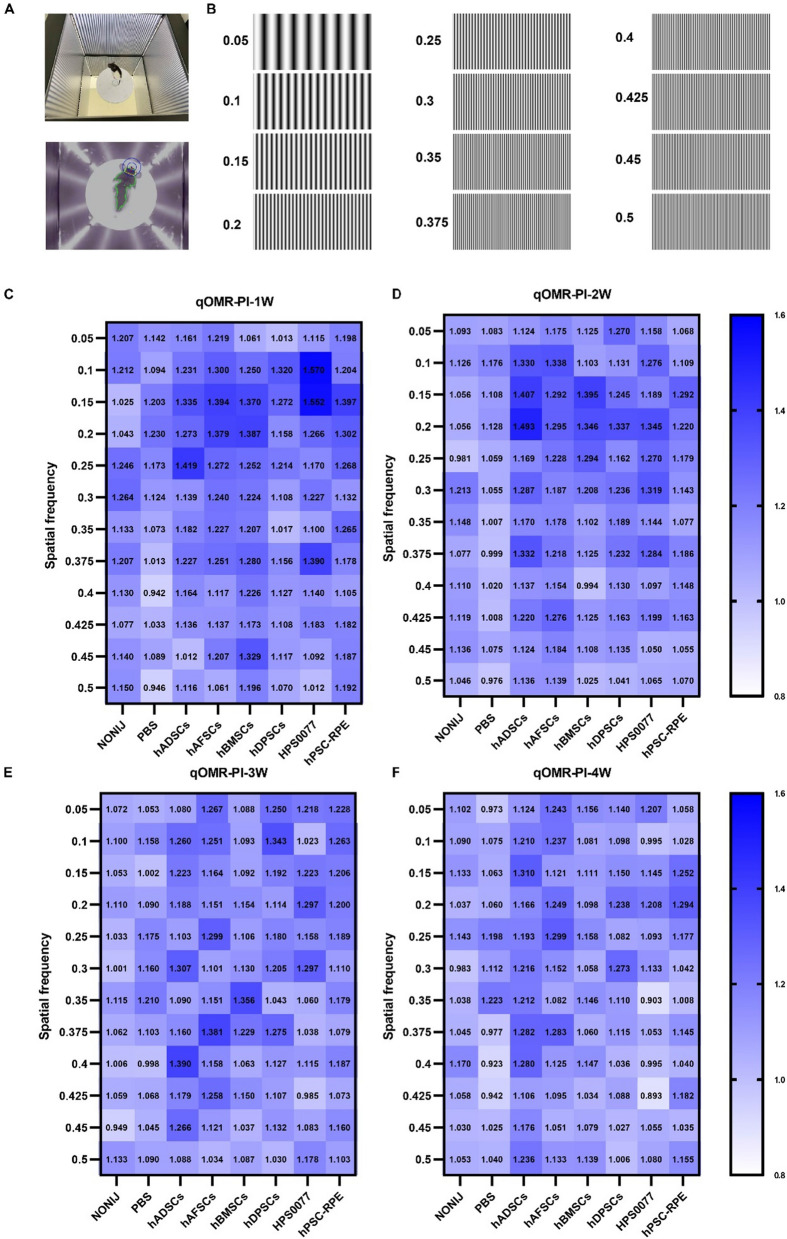
Visual function of RCS rats determined by the quantitative optomotor response (qOMR) in groups subjected to subretinal injection of different cells. **A** Photos of RCS rats used to evaluate the qOMR. **B** A range of stimuli at different spatial frequencies were used, including 0.05, 0.1, 0.15, 0.2, 0.25, 0.3, 0.35, 0.375, 0.4, 0.425, 0.45, and 0.5 cycles/degree with movement at 12 degree/s for 60 s. The smaller the value of the spatial frequency, the greater the spacing of the black and white stripes is. **C**–**F** Heatmap of the average qOMR index values of different groups of RCS rats, which were subjected to subretinal transplantation or not injected with cells or PBS, at 1 week (**C**), 2 weeks (**D**), 3 weeks (**E**), and 4 weeks (**F**) post-injection. The higher the qOMR index is, the color is more blue. The lower the qOMR index is, the color is more white

The qOMR system, which was equipped with four screens on each wall of the box, was used to evaluate the visual function of RCS rats subjected to subretinal transplantation of several types of stem cells and hiPSC-RPE cells at 1 to 4 weeks post-injection (Fig. 5A) (Additional file 3: video S2). Unlike human, animals are hard to describe how their visions are. The qOMR evaluation is an equipment with rotating stripes that animals are willing to follow can help determination of the visual behavior of animals. The qOMR evaluation of RCS rats was conducted randomly at several different spatial frequencies (0.05, 0.1, 0.15, 0.2, 0.25, 0.3, 0.35, 0.375, 0.4, 0.425, 0.45, and 0.5 cycles/degree) (Fig. 5B). Spatial frequency refers to the number of grid cycles in which the light and dark components of the image or stimulus pattern are sinusoidally modulated in each degree of the viewing angle, and the unit is cycles per degree. The qOMR index, which was defined according to the correct/incorrect tracking behaviors of the RCS rats, was monitored and calculated automatically using qOMR tracking algorithms. The higher the qOMR index is, usually the better the visual function of RCS rats is.

The qOMR index of each group at 1, 2, 3, and 4 weeks post-injection of the cells is shown in heatmaps in Fig. 5C–F, with a higher qOMR index value shown in darker blue and a lower qOMR index value shown in a lighter blue, which is close to white. The qOMR index decreased at 3 weeks and 4 weeks post-injection compared to that at 1 week and 2 weeks post-injection in all groups. A higher qOMR index value, with darker blue in the heatmaps, was found in the cell transplantation groups (hADSCs: 1.273 ± 0.296, hAFSCs: 1.379 ± 0.300, hBMSCs: 1.387 ± 0.355, hDPSCs: 1.158 ± 0.168, hiPSCs: 1.266 ± 0.179, hiPSC-RPE cells: 1.302 ± 0.179) than in the control groups (NONIJ: 1.043 ± 0.231, PBS: 1.23 ± 0.197) at 1 week post-injection, when the spatial frequency was 0.2 (Fig. 5C). Similar results were also obtained over the following 3 weeks (2, 3, and 4 weeks). Compared to that of the control group (NONIJ: 1.056 ± 0.246, PBS: 1.128 ± 0.285), a higher qOMR index value was found for the cell transplantation groups (hADSCs: 1.493 ± 0.318, hAFSCs: 1.295 ± 0.179, hBMSCs: 1.346 ± 0.325, hDPSCs: 1.337 ± 0.290, hiPSCs: 1.345 ± 0.169, hiPSC-RPE cells: 1.220 ± 0.239) at 2 weeks post-injection (spatial frequency: 0.2) (Fig. 5D). At 3 weeks and 4 weeks post-injection, the cell transplantation groups [hADSCs: 1.188 ± 0.261 (3 weeks), 1.166 ± 0.112 (4 weeks), hAFSCs: 1.151 ± 0.133 (3 weeks), 1.249 ± 0.226 (4 weeks), hBMSCs: 1.154 ± 0.210 (3 weeks), 1.098 ± 0.208 (4 weeks), hDPSCs: 1.114 ± 0.168 (3 weeks), 1.238 ± 0.198 (4 weeks), hiPSCs: 1.297 ± 0.134 (3 weeks), 1.208 ± 0.403 (4 weeks), hiPSC-RPE cells: 1.2 ± 0.162 (3 weeks), 1.294 ± 0.168 (4 weeks)] still showed higher qOMR index values than the control groups [NONIJ: 1.11 ± 0.182 (3 weeks), 1.03 ± 0.201 (4 weeks), PBS: 1.09 ± 0.149 (3 weeks), 1.06 ± 0.139 (4 weeks)] when the spatial frequency was 0.2. (Spatial frequency: 0.2) (Fig. 5E, F). The qOMR results indicated that the visual behavior of RCS rats improved slightly in the cell transplantation groups compared to the age-matched control groups at a specific frequency of 0.2 from 1 to 4 weeks post-injection.

### Visual function evaluation of RCS rats by ERG measurement

**Fig. 6:**
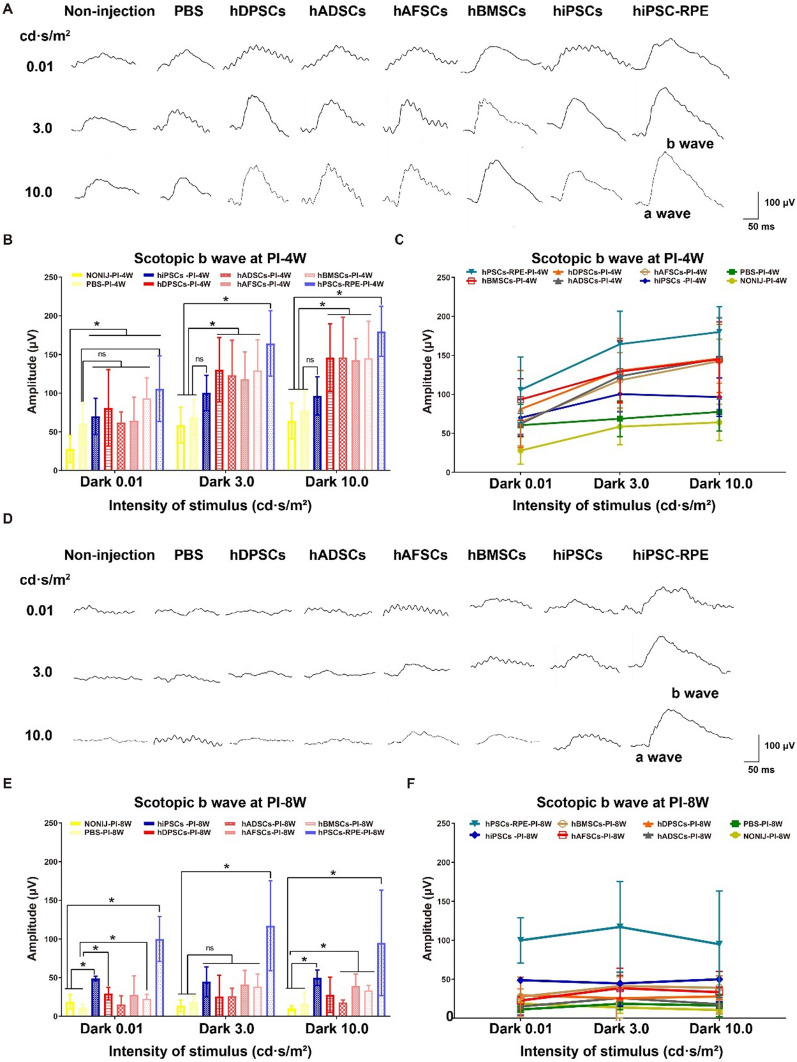
Visual function detected by electroretinogram (ERG) in different groups of RCS rats after subretinal cell injection. **A** Representative dark-adapted ERG performance at 0.01, 3.0, and 10.0 cd s/m^2^ intensities for different groups of RCS rats at 4 weeks post-injection: non-cell injection (NONIJ, age-matched control), PBS injection, and cell injection of hDPSCs, hADSCs, hAFSCs, hBMSCs, hiPSCs, and hiPSC-RPE cells. The higher the amplitude of the wave is, the better the visual function is. **B** The average amplitude of scotopic b waves at 0.01, 3.0, and 10.0 cd·s/m^2^ intensities for RCS rats in different groups at 4 weeks post-injection: non-cell injection (NONIJ, age-matched control), PBS injection, and cell injection of hDPSCs, hADSCs, hAFSCs, hBMSCs, hiPSCs, and hiPSC-RPE cells. RCS rats subjected to four kinds of hMSCs or hiPSC-RPE cells transplantation showed a significant higher amplitude of the wave at 4 weeks post-injection. **C** Line graph of the average amplitude of scotopic b wave at 0.01, 3.0, and 10.0 cd s/m^2^ intensities for RCS rats in different groups at 4 weeks post-injection: non-cell injection (NONIJ, age-matched control), PBS injection, and cell injection of hDPSCs, hADSCs, hAFSCs, hBMSCs, hiPSCs, and hiPSC-RPE cells. **D** Representative dark-adapted ERG performance at 0.01, 3.0, and 10.0 cd s/m^2^ intensities for RCS rats in different groups at 8 weeks post-injection: non-cell injection (NONIJ, age-matched control), PBS injection, and cell injection of hDPSCs, hADSCs, hAFSCs, hBMSCs, hiPSCs, and hiPSC-RPE cells. **E** The average amplitude of scotopic b waves at 0.01, 3.0, and 10.0  cd s/m^2^ intensities for RCS rats in different groups at 8 weeks post-injection: non-cell injection (NONIJ, age-matched control), PBS injection, and cell injection of hDPSCs, hADSCs, hAFSCs, hBMSCs, hiPSCs, and hiPSC-RPE cells. Only RCS rats subjected to hiPSC-RPE cells transplantation showed a significant higher amplitude of the wave at 8 weeks post-injection. **F** Line graph of the average amplitude of scotopic b wave at 0.01, 3.0, and 10.0 cd s/m^2^ intensities for RCS rats in different groups at 8 weeks post-injection: non-cell injection (NONIJ, age-matched control), PBS injection, and cell injection of hDPSCs, hADSCs, hAFSCs, hBMSCs, hiPSCs, and hiPSC-RPE cells. *p < 0.05; “ns”: the difference was not statistically significant

Scotopic electroretinography waves of age-matched RCS rats that were noninjected, subjected to sham transplantation of PBS, or subjected to transplantation of different cells (stem cells and hiPSC-RPE cells) were recorded to evaluate visual function at 4 weeks and 8 weeks post-injection (Additional file 1: Fig. S3). The ERG b wave was measured from the trough of the first negative wave to the peak of the first positive wave (Fig. 6A). Compared to age-matched control rats, RCS rats that received any transplanted cells exhibited a stronger ERG response at a standard stimulus for the scotopic ERG at 4 weeks post-injection at any stimulus intensity (0.01, 3.0 and 10.0 cd s/m^2^) (Fig. 6A). Among the groups, RCS rats transplanted with hiPSC-RPE cells exhibited the strongest b-wave responses, which indicated the highest potential for preservation of visual function (Fig. 6B, C). In contrast, RCS rats that received hiPSCs showed a limited ERG response compared to that of RCS rats that received hiPSC-RPE cells or hMSCs (hDPSCs, hADSCs, hAFSCs, and hBMSCs) (Fig. 6B, C). On the other hand, hMSC transplantation in RCS rats also showed a promising ability to maintain the ERG response at 4 weeks post-injection compared to that of the control groups (sham-injected PBS injection group and noninjected RCS rat group) (*p* < 0.05), which manifested the transplantation of hDPSCs, hADSCs, hAFSCs, or hBMSCs was effective in vision restoration at 4 weeks post-injection (Fig. 6B and C). In addition, the ERG responses of RCS rats in the four hMSC (hDPSC, hADSC, hAFSC, and hBMSC) transplantation groups were similar (*p* > 0.05), which indicated the nearly effectiveness of both fetal stem cells and adult stem cells (Fig. 6B and C).

To avoid the influence of cell sources, we also compared the protective effects of four different hAFSCs derived from four different (independent) donors. No significant differences in the ERG responses of RCS rats were found among the primary hAFSCs from four different donor sources after transplantation in this study (*p* > 0.05) (Additional file 1: Fig. S3). These results indicated that there was no significant influence of the cell source of specific types of primary cells on the visual function of RCS rats that received stem cells from different donor sources in this study.

The apoptosis of photoreceptor cells was reported to begin at postnatal week 3 in RCS rats, and almost all photoreceptor cells die in 2- (8-week-old) to 3-month-old RCS rats [37, 45, 46]. Therefore, we transplanted each cell type into RCS rats at the beginning of the period of apoptosis of photoreceptor cells [postnatal day 21 (3 weeks)] and detected the second ERG response at week 8 postinjection, when the RCS rats were 11 weeks old. The results are shown in Fig. 6D–F. Almost no ERG responses of RCS rats were recorded in the age-matched control groups (untreated group and sham-injected PBS group) at week 8 post-injection (Fig. 6D). Similarly, almost no ERG responses of RCS rats were observed in RCS rats that received hMSCs (hDPSCs, hADSCs, hAFSCs, and hBMSCs) or hiPSCs at week 8 post-injection (Fig. 6D). Surprisingly, distinct ERG responses were observed in RCS rats that received hiPSC-RPE cells at 8 weeks post-injection (Fig. 6D). Quantitatively, ERG responses were sharply reduced in most of the RCS rats that received hMSCs or hiPSCs as well as the age-matched control groups (the untreated group and sham-injected PBS group), except for those in RCS rats that received hiPSC-RPE cells, at week 8 post-injection (Fig. 6E, F). These results indicated that RCS rats subjected to transplantation of hiPSC-RPE cells but not RCS rats subjected to transplantation of hMSCs or hiPSCs, maintained their visual function at week 8 post-injection. The effect of subretinal hMSC transplantation in RCS rats is relatively short (up to 4 weeks post-injection), whereas the effect of subretinal hiPSC-RPE cell transplantation in RCS rats lasts longer (more than 8 weeks post-injection).

### Histological analysis of retinal structures in RCS rats after transplantation of several cell types

**Fig. 7:**
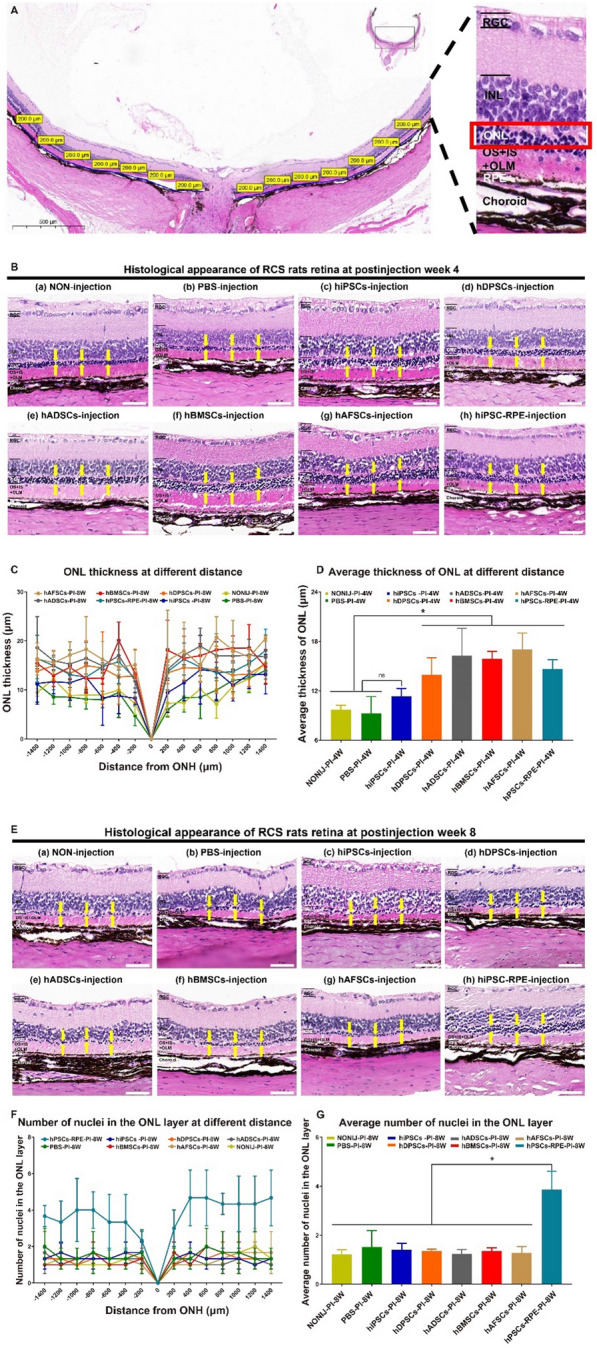
Histological evaluation of the retinas of RCS rats subjected to subretinal transplantation of several different types of cells. **A** Representative hematoxylin–eosin (HE) staining image of the retinas of RCS rats, in which 14 points at the same interval were selected to evaluate the outer nuclear layer (ONL). *RGC* retinal ganglion cell, *INL* inner nuclear layer, *OLM* outer limited membrane, *OS* outer segment, *IS* inner segment, *RPE* retinal pigment epithelium. **B** Representative histological analysis of the retinas of RCS rats that received no injection (noninjection) (a) and RCS rats that received a subretinal injection of PBS (b), hiPSCs (c), hDPSCs (d), hADSCs (e), hBMSCs (f), hAFSCs (g), or hiPSC-RPE cells (h) at 4 weeks post-injection. The ONL is indicated with yellow arrows. The thicker the ONL is, the greater the number of the cell body of photoreceptor cells is. **C** The ONL thickness at 14 points for RCS rats that received no injection (noninjection) and RCS rats that received a subretinal injection of PBS, hiPSCs, hDPSCs, hADSCs, hBMSCs, hAFSCs, or hiPSC-RPE cells at 4 weeks post-injection. **D** Average thickness of the ONL at 14 points for RCS rats that received no injection (noninjection) and RCS rats that received a subretinal injection of PBS, hiPSCs, hDPSCs, hADSCs, hBMSCs, hAFSCs, or hiPSC-RPE cells at 4 weeks post-injection. RCS rats subjected to four kinds of hMSCs or hiPSC-RPE cells transplantation showed a significant thicker ONL at 4 weeks post-injection. **E** Representative histological appearance of retinas from RCS rats that received no injection (noninjection) (a) and RCS rats that received a subretinal injection of PBS (b), hiPSCs (c), hDPSCs (d), hADSCs (e), hBMSCs (f), hAFSCs (g), or hiPSC-RPE cells (h) at 8 weeks post-injection. The ONL is indicated by yellow arrows. **F** The number of nuclei in the ONL at 14 points for RCS rats that received no injection (noninjection) and RCS rats that received a subretinal injection of PBS, hiPSCs, hDPSCs, hADSCs, hBMSCs, hAFSCs, or hiPSC-RPE cells at 8 weeks post-injection. **G** Average number of nuclei in the ONL at 14 points for RCS rats that received no injection (noninjection) and RCS rats that received a subretinal injection of PBS, hiPSCs, hDPSCs, hADSCs, hBMSCs, hAFSCs, or hiPSC-RPE cells at 8 weeks post-injection. Only RCS rats subjected to hiPSC-RPE cells transplantation showed a significant thicker ONL at 8 weeks post-injection. *p < 0.05; “ns”: the difference was not statistically significant

Histological analysis of the retina was conducted to evaluate the differences in the retinal structure of RCS rats subjected to transplantation with several types of cells and rats that did not receive an injection (control group). The cell body of photoreceptor cells is located in the retinal ONL, which becomes thinner with the progression of retinal degeneration in RCS rats or patients with retinal degeneration, including patients with AMD and Stargardt’s macular dystrophy. Therefore, evaluations of the ONL of RCS rats at 14 different points, which were set every 200 μm from the optic nerve head, were conducted at week 4 and week 8 post-injection in this study (Fig. 7A). The thickness of the ONL was greater in RCS rats subjected to transplantation with stem cells (hMSCs (hAFSCs, hADSCs, hBMSCs, and hDPSCs) and hiPSCs) or hiPSC-RPE cells than in the control rats (rats in the untreated group and sham-injected PBS group) at week 4 post-injection (*p* < 0.05) (Fig. 7B), which confirmed the preservation of retinal structure and retinal thickness after cell transplantation. In addition, the ONL thickness of RCS rats in the cell transplantation groups at 4 weeks post-injection was almost the same at all 14 time points (*p* > 0.05) (Fig. 7C). The groups that received hiPSC-RPE cells and four hMSCs (hADSCs, hAFSCs, hBMSCs, and hDPSCs) showed similar preservation of ONL thickness, with greater thickness than RCS rats subjected to transplantation with hiPSCs and control rats based on the analysis of average ONL thickness (*p* < 0.05) (Fig. 7D).

Figures 7E–G show the histological analysis of the retina (Fig. 7E) and the number of nuclei per column in the ONL layer in RCS rats subjected to transplantation with several types of cells at 8 weeks post-injection (Fig. 7F and G). ONL thickness was difficult to measure in RCS rats that received hMSCs and hiPSCs as well as RCS rats in the control group at 8 weeks post-injection. Therefore, the number of nuclei per column in the ONL layer in the direction perpendicular to the ONL layer was evaluated instead of ONL thickness at 8 weeks post-injection. The ONL thickness was smaller in RCS rats subjected to transplantation with hMSCs and hiPSCs as well as those that did not receive any cells (control group) at 8 weeks post-injection (Fig. 7E). However, RCS rats that received hiPSC-RPE cells showed a higher number of nuclei per column in the ONL at all 14 measurement points than RCS rats that received other cell types or no cells (control groups) (*p* < 0.01) (Fig. 7F). There were found to be approximately 4 nuclei per column in the ONL on average in RCS rats in the hiPSC-RPE cell transplantation group, which was higher than the numbers in the other cell transplantation groups and the control groups (*p* < 0.05), where the number of nuclei per column was approximately 1 (Fig. 7G). The results indicated that only RCS rats subjected to hiPSC-RPE transplantation preserved their retinal structure and retinal thickness at 8 weeks post-injection.

The histological analysis showed that the subretinal transplantation of hADSCs, hAFSCs, hBMSCs, hDPSCs, or hiPSCs into RCS rats resulted in temporary preservation of the retinal ONL for up to 4 weeks, whereas the subretinal transplantation of hiPSC-RPE cells into RCS rats resulted in long-term preservation of the retinal ONL for at least 8 weeks. The results obtained by histological analysis were found to be consistent with the results obtained by ERG analysis.

## Discussion

Stem cell-based therapy is promising for RD patients. However, which stem cell types have better protective effects was unknown until this investigation. In this study, subretinal transplantation of hiPSC-RPE cells showed a better and longer effect than transplantation of hADSCs, hAFSCs, hBMSCs, hDPSCs, and hiPSCs in terms of protecting both visual function and retinal structure in an RD animal model in RCS rats. hADSCs, hAFSCs, hBMSCs, and hDPSCs yielded better preservation of the retinal structure and function than hiPSCs (HPS0077), although the effect was temporary, lasting until 4 weeks post-transplantation but not 8 weeks post-transplantation. Overall, hiPSC-RPE cells showed the best protective effect; hADSCs, hAFSCs, hBMSCs, and hDPSCs had the next best protective effect; and hiPSCs (HPS0077) had the weakest protective effect. Our study investigated the protective effect of each type of stem cell and performed a comparison in RCS retinas, and the results laid a foundation for future research aimed at optimizing stem cell-based therapies for RD and possibly other degenerative diseases.

Every cell line used in this study was validated using flow cytometry or immunostaining to confirm they are qualified cells with the expression of specific markers before subretinal transplantation. Our results confirmed the specific expression of the hMSC markers CD44, CD73, and CD105 in four types of hMSCs (hAFSCs, hADSCs, hDPSCs, and hBMSCs), as well as the lack of expression of the human hematopoietic progenitor marker CD34 [48]. In addition, the human pluripotent markers Nanog, OCT4, and SSEA4 were detected in the hiPSC cell line HPS0077. The RPE cells used in this study were differentiated from hiPSCs (HPS0077), and they are referred to as hiPSC-RPE cells. These hiPSC-RPE cells exhibited the characteristic polygonal morphology and produced dark pigments, similar to native mature RPE cells [55, 56]. In addition, the previously reported RPE-related markers MITF, PAX6, RPE65, and ZO-1 were all highly expressed in our hiPSC-RPE cells [35, 37, 57, 58]. Although the validation of cells was carried out using specific markers expression, the cellular function and the purity of differentiated cells should be confirmed before real clinical treatments in future.

Lentivirus-GFP was reported to label RPE cells for detection in vivo [34]. Therefore, we tried to label stem cells such as hAFSCs and hDPSCs using lentivirus-GFP. We observed green fluorescence from hAFSCs and hDPSCs in vitro under the microscope after transduction of lentivirus-GFP into the cells, but the green fluorescence was found to be extremely weak in the eyes of RCS rats by fundus photography in vivo after subretinal transplantation of the cells into RCS rats. In addition, a potential risk of changing cellular processes or gene expression by the use of transgenic markers GFP still exists. CellTracker was also previously reported for the detection of RPE cells in vivo by several researchers [37, 42] and is considered safer than lentivirus-GFP transduction. Our results demonstrated that CellTracker staining of the cells was suitable for labeling and observation of all six cell lines, namely, hiPSC-RPE, hAFSCs, hADSCs, hBMSCs, hDPSCs, and hiPSCs, in vivo. The green fluorescence in fundus photographs was evidence of successful transplantation of the cells into the subretinal space. Although we have proofed transplanted cells were successful transplanted into the subretinal space by the green fluorescence of CellTraker, a long-lasting cell tracker which can trace cells for months or years would be beneficial for the evaluation of cell integration and survival in vivo in future.

In RCS rats, a tendency to follow rotating stripes (qOMR) or a preference for the dark (LDB) can partially reflect visual function [59, 60], although the reliability of the results is influenced by involuntary movements of the rats, such as chewing their digits or licking their fur. Our results showed an increase in qOMR index values at most of the spatial frequencies in groups subjected to transplantation of all six cell lines compared with the RCS rat group subjected to PBS injection or the age-matched noninjection group from week 1 to week 4 post-injection. In addition, most of the higher qOMR values appeared at a spatial frequency of approximately 0.2, which was consistent with previous studies in which animals showed the best performance following the stripes at the same spatial frequency of 0.2 [51]. Although the qOMR index values were improved in the RCS rats subjected to transplantation of cells, no significant difference in the duration of time spent in the light and dark chambers was found in LDB testing under natural light conditions. Because the RCS rats had RD and impaired vision, light of a strong intensity should be used for irradiation of the light chamber rather than natural light conditions to induce more significant differences between the light and dark chambers. Moreover, other behavioral assays assisting in visual function evaluation, such as a watermaze assay, should also take into consideration in the further study.

ERG is an objective visual electrophysiological examination that is considered the gold standard for evaluating the visual function of the retina [61, 62]. The ERG performance of RCS rats decreased dramatically with the progression of RD [42]. In our results, we observed the preservation of ERG performance to varying degrees in the groups subjected to transplantation of all six cell lines (the hiPSC-RPE group had slightly higher performance than the four hMSC groups (the hAFSC, hADSC, hBMSC, and hDPSC groups) and much higher performance than the hiPSC group, the PBS injection group or the age-matched noninjection group at 4 weeks post-injection. However, only the hiPSC-RPE group maintained partial ERG performance at 8 weeks post-injection, which indicated that hiPSC-RPE cells induced better and longer protection of visual function than other cells, whereas hAFSCs, hADSCs, hBMSCs, hDPSCs, and hiPSCs induced temporary protection that lasted only until 4 weeks.

The photoreceptor cells in the retina of RCS rats began to undergo apoptosis from postnatal week 3 and were nearly completely lost when the rats were 2 to 3 months of age [45, 46]. The cell body of photoreceptor cells lies in the ONL of the retina, and a thicker ONL or a greater number of nuclei per column in the ONL indicates the existence of more photoreceptor cells. In our results, hiPSC-RPE cells led to the preservation of approximately 4 nuclei per column in the ONL and slowed the progression of retinal degeneration even at 8 weeks post-injection; at this time, the RCS rats were 2 to 3 months old, and the most severe loss of photoreceptor cells was expected in nontreated RCS rats. Other types of cells, such as hAFSCs, hADSCs, hBMSCs, hDPSCs, and hiPSCs, preserved the thickness of the ONL only at week 4 post-injection but not at week 8 post-injection.

The results of retinal histological analysis were also consistent with the above visual function results obtained by ERG. It is known that the well-organized retinal structure is the foundation of the normal visual function. Photoreceptor cells lies in the ONL are responsible for the photoreception and phototransduction, which are crucial for vision. Stem cell-based transplantation preserved the visual function detected by ERG with a result of maintaining the thickness of ONL, which was evaluated by the histological analysis.

hAFSCs are fetal stem cells and have been reported to have stronger differentiation abilities than other hMSCs, such as hBMSCs and hADSCs [63]. hDPSCs have also been reported to exhibit higher proliferation than other hMSCs, such as hBMSCs and hADSCs [64]. However, we did not observe differences among different types of hMSCs in the protection of visual function or ONL thickness subretinal transplantation into RCS rats.

Consistent with the previous studies [33, 37, 52, 65], RCS rats or other animal models of RD, which underwent a progressive retinal degeneration, can be halted by stem cell-based therapy. Our study confirmed the protective effect of different stem cells and hiPSC-RPE cells on RCS rats with various degrees, and figured out the most effective stem cell-based therapy for RD that is the subretinal transplantation of hiPSC-RPE cells. It is promising to treat patients with RD or other degenerative diseases by transplantation of hiPSC-RPE cells or other regenerative cells to recover and maintain cell function in future. However, there are still some challenges should be considered seriously. For instance, the purity of stem cell derived cells and the long-term safety of transplanting those cells should be assessed carefully [17]; a world recognized and standard protocol to prepare and transplant cells should be established; a potential cellular rejection after cell transplantation also should take into consideration; the ethical and regulatory aspects related to stem cell-based therapy should be treated seriously [66].

We speculated that the protective effect of subretinal transplantation of these cells against RD in RCS rats partially relied on the trophic factors that the cells secreted [67] and the cell fusion and materials transfer between the transplanted stem cells and the native cells. Growth factors or pathways related to GDNF [68, 69], BDNF [70, 71], PEDF [72, 73], VEGF [74, 75], and TGF-β [76] were reported to have neuroprotective effects. We observed the secretion of three growth factors, GDNF, TGF-β, and BDNF, by ELISA in all six cell lines we transplanted. However, the secretion of PEDF and VEGF was observed only in hiPSC-RPE cells. A more detailed and comprehensive analysis of the trophic factors of the transplanted cells secreted in vivo, using microarray or RNA expression assay may contribute to a better understanding of the molecular mechanisms in future. In addition, we speculated that another reason why hiPSC-RPE cells had the strongest retinal protection ability in RCS rats was that hiPSC-RPE cells may replace partially dysfunctional native RPE cells, which needs to be confirmed in further studies. For instance, the integration of transplanted cells with native cells should be confirmed by using an electron microscopy, and the function of transplanted cells should be evaluated in vivo.

Although we confirmed that hiPSC-RPE cell transplantation showed the best and longest protective effect in the retinas of RCS rats compared to the transplantation of other cells investigated in this study, such as hAFSCs, hADSCs, hBMSCs, hDPSCs, and hiPSCs, which also rescued the visual function of RCS rats, but the effect was temporary and lasted only 4 weeks. Besides, the potential of hESCs, hESCs-RPE cells, and hiPSC-MSC cells in RD treatment still needs to be investigated and compared in future. In addition, we confirmed the protective effect of subretinal transplantation of these cells against RD in RCS rats partially relied on the secretion of growth factors by the cells. hMSCs and hiPSCs as well as hiPSC-RPE cells can secrete GDNF, BDNF, and TGF-β. hiPSC-RPE cells can secrete the growth factors PEDF and VEGF, which cannot be secreted by hMSCs or hiPSCs. However, there are still some limitations need to be further investigated. First, it should be considered how long each cell can survive in the eyes of RCS rats after subretinal transplantation by long-term follow-up experiments using cell tracker on the retinal section. Second, it should be more specifically explored and compared how is the protective mechanism of hESCs, hESCs-RPE cells, or hiPSC-MSC cells on RD progress of RCS rats by combining the microarray assays and cell integration detection using electron microscope. Third, it needs to be explored whether the number of transplanted cells or numbers of injection time give influence on the treatment outcome by setting up a series of gradient cell concentrations or injection frequency for transplantation. Furthermore, the methods chosen to deliver cells may also influence the outcomes. For instance, cell sheet transplantation kept the integrity and well-aligned structure of cells, but showed large surgical trauma without obvious vision improvement in clinical patients [77, 78]; transplantation of cell suspension showed some effect, but with the risk of backflow. Therefore, injectable hydrogels loaded cell transplantation should be an alternative delivery method for patients in the future by loading cells on injectable hydrogels, which can be solidified in situ at physiological condition. In addition, immunosuppression drugs were used to avoid cell rejection in this study. How to avoid the usage of immunosuppression drugs will benefit patients who will receive cell transplantation, which may include the personalized therapy using self-origin cells from patients or generate regenerative cells from hypo-immunogenic stem cells.

### Supplementary Information

**Additional file 1: Table S1.** Materials used in this study. **Fig. S1.** Subretinal injection and characterization. **Fig. S2.** Exclusion criterion. **Fig. S3.** Electroretinogram performance.

**Additional file 2: Movie S1.** Light-dark box assay on RCS rats.

**Additional file 3: Movie S2.** Optomotor response assay on RCS rats.

## Data Availability

All relevant data supporting the key findings of this study are available within the article and its Supplementary Information files or from the corresponding author upon reasonable request.

## Abbreviations

RPE: Retinal pigment epithelium
RD: Retinal degeneration
hiPSC: Human induced pluripotent stem cell
RCS: Royal College of Surgeons
OMR: Optomotor response
ERG: Electroretinography
HE: Hematoxylin and eosin
RP: Retinitis pigmentosa
AMD: Age-related macular degeneration
FDA: Food and Drug Administration
VEGF: Vascular endothelium growth factor
hBMSCs: Human bone marrow stem cells
hDPSCs: Human dental pulp stem cells
hADSCs: Human adipose-derived stem cells
hAFSCs: Human amniotic fluid stem cells
hESCs: Human embryonic stem cells
*Mertk*: MER proto-oncogene, tyrosine kinase
LDB: Light–dark box
DM: Differentiation medium
KSR: KnockOut serum replacement
NEAA: MEM nonessential amino acids
NIC: Nicotinamide
CTM: Chetomin
PFA: Paraformaldehyde
ELISA: Enzyme-linked immunosorbent assay
BDNF: Brain-derived neurotrophic factor
GDNF: Glial cell line-derived neurotrophic factor
PEDF: Pigment epithelium derived factor
TGF-β: Transforming growth factor-β
HGF: Hepatocyte growth factor
FGF2: Fibroblast growth factor 2
GFP: Green fluorescent protein
ONL: Outer nuclear layer
ONH: Optic nerve head
