# Supplementary material for: Comparison of retinal degeneration treatment with four types of different mesenchymal stem cells, human induced pluripotent stem cells and RPE cells in a rat retinal degeneration model
Source: J Transl Med. 2023 Dec 14;21:910. doi: 10.1186/s12967-023-04785-1 (PMC10720187; doi:10.1186/s12967-023-04785-1)
Supplement: Supplementary file 1 — Additional file 1: Table S1. Materials used in this study. Fig. S1. Subretinal injection and characterization. Fig. S2. Exclusion criterion. Fig. S3. Electroretinogram performance. [file 12967_2023_4785_MOESM1_ESM.docx]

**Comparison of retinal degeneration treatment with four types of different mesenchymal stem cells, human induced pluripotent stem cells and RPE cells in a rat retinal degeneration model**

**Authors:** Qian Liu^1^, Jun Liu^1^, Minmei Guo^1^, Tzu-Cheng Sung^1^, Ting Wang^1^, Tao Yu^1^, Zeyu Tian^1^, Guoping Fan^2^, Wencan Wu^1^, Akon Higuchi^1,3*^

**Affiliations:**

^1^State Key Laboratory of Ophthalmology, Optometry and Visual Science, Eye Hospital, Wenzhou Medical University; Wenzhou, China.

^2^Department of Human Genetics, David Geffen School of Medicine UCLA; Los Angeles, USA.

^3^Department of Chemical and Materials Engineering, National Central University; Taiwan, China.

*Corresponding author. Email: higuchi@ncu.edu.tw & [higuchi@wmu.edu.cn](mailto:higuchi@wmu.edu.cn)).

**List of Supplementary Materials**

Table S1. Materials used in this study.

Fig. S1. Subretinal injection and characterization.

Fig. S2. Exclusion criterion.

Fig. S3. Electroretinogram performance.

Movie S1. Light-dark box assay on RCS rats.

Movie S2. Optomotor response assay on RCS rats.

Additional file Table

| **Table S1** Materials used in this study. | | | |
| --- | --- | --- | --- |
| Materials | Abbreviation | Catalog No. | Company |
| **Cell culture medium and component** | | | |
| Primary mesenchymal stem cell culture medium | MSC medium | PriMed-iCell-012 | iCellbioscience (Shanghai, China) |
| mTESR1 medium | mTESR1 | 85850 | Stemcell Technologies  (Vancouver, Canada) |
| DMEM/F12 medium | DMEM/F12 | 11330-057 | Thermo Fisher Scientific Inc. (Waltham, MA, USA) |
| DMEM | DMEM | 11965 | Thermo Fisher Scientific Inc. (Waltham, MA, USA) |
| F12 | F12 | 11765 | Thermo Fisher Scientific Inc. (Waltham, MA, USA) |
| KnockOut Serum Replacement | KSR | 10828 | Thermo Fisher Scientific Inc. (Waltham, MA, USA) |
| MEM nonessential amino acids | NEAA | 11140 | Thermo Fisher Scientific Inc. (Waltham, MA, USA) |
| Glutamine | glutamine | 25030 | Thermo Fisher Scientific Inc. (Waltham, MA, USA) |
| Antibiotic-antimycotic | anti-anti | 15240 | Thermo Fisher Scientific Inc. (Waltham, MA, USA) |
| β-mercaptoethanol | β-mercaptoethanol | M3148 | Sigma-Aldrich (St. Louis, MO, USA) |
| Chetomin | CTM | C9623 | Sigma-Aldrich (St. Louis, MO, USA) |
| B-27™ Supplement | B27 | 17504044 | Thermo Fisher Scientific Inc. (Waltham, MA, USA) |
| **Antibodies** | | | |
| FITC-labeled anti-CD44 | anti-CD44 | 11-0441-82 | Thermo Fisher Scientific Inc. (Waltham, MA, USA) |
| PE-labeled anti-CD73 | anti-CD73 | 12-0739-42 | Thermo Fisher Scientific Inc. (Waltham, MA, USA) |
| PE-labeled anti-CD105 | anti-CD105 | 12-1057-42 | Thermo Fisher Scientific Inc. (Waltham, MA, USA) |
| FITC-labeled anti-CD34 | anti-CD34 | 11-0349-41 | Thermo Fisher Scientific Inc. (Waltham, MA, USA) |
| FITC-labeled isotype | Isotype-FITC | 11-471482 | Thermo Fisher Scientific Inc. (Waltham, MA, USA) |
| PE-labeled isotype | Isotype-PE | 12-4714-82 | Thermo Fisher Scientific Inc. (Waltham, MA, USA) |
| anti-MiTF | anti-MiTF | ab3201 | Abcam (Cambridge, UK) |
| anti-PAX6 | anti-PAX6 | MA532409 | Thermo Fisher Scientific Inc. (Waltham, MA, USA) |
| anti-RPE65 | anti-RPE65 | MA116578 | Thermo Fisher Scientific Inc. (Waltham, MA, USA) |
| anti-ZO1 | anti-ZO1 | 402200 | Thermo Fisher Scientific Inc. (Waltham, MA, USA) |
| mouse isotype antibodies | Isotype-mouse | ab81216 | Abcam (Cambridge, UK) |
| rabbit isotype antibodies | Isotype-rabbit | ab172730 | Abcam (Cambridge, UK) |
| anti-mouse IgG H&L FITC | FITC | ab6785 | Abcam (Cambridge, UK) |
| anti-rabbit IgG H&L PE | PE | ab72465 | Abcam (Cambridge, UK) |
| anti-Nanog | anti-Nanog | MA1017 | Thermo Fisher Scientific Inc. (Waltham, MA, USA) |
| anti-OCT4 | anti-OCT4 | PA527438 | Thermo Fisher Scientific Inc. (Waltham, MA, USA) |
| anti-SSEA4 | anti-SSEA4 | MA1021 | Thermo Fisher Scientific Inc. (Waltham, MA, USA) |
| goat anti-rabbit IgG H&L Alexa Fluor 488 | goat anti-rabbit IgG H&L Alexa Fluor 488 | ab150077 | Abcam (Cambridge, UK) |
| goat anti-mouse IgG H&L Alexa Fluor® 594 | goat anti-mouse IgG H&L Alexa Fluor® 594 | ab150116 | Abcam (Cambridge, UK) |
| **EILSA kit** | | | |
| Human BDNF-ELISA kit | BDNF | JL11683 | Jianglaibio (Shanghai, China) |
| Human GDNF-ELISA kit | GDNF | JL12988 | Jianglaibio (Shanghai, China) |
| Human PEDF-ELISA kit | PEDF | JL10799 | Jianglaibio (Shanghai, China) |
| Human VEGF-ELISA kit | VEGF | JL18341 | Jianglaibio (Shanghai, China) |
| Human TGF-β-ELISA kit | TGF-β | JL20082 | Jianglaibio (Shanghai, China) |
| Human HGF-ELISA kit | HGF | JL10756 | Jianglaibio (Shanghai, China) |
| Human FGF2-ELISA kit | FGF2 | JL14546 | Jianglaibio (Shanghai, China) |
| **Other materials** | | | |
| Matrigel | Matrigel | 356230 | Corning (Corning, NY, USA) |
| Trypsin-EDTA phenol red | trypsin-EDTA | 25200 | Thermo Fisher Scientific Inc. (Waltham, MA, USA) |
| Triton X-100 | Triton X-100 | P0096 | Beyotime (Shanghai, China) |
| Blocking buffer | blocking buffer | P0260 | Beyotime (Shanghai, China) |
| CellTracker Green probe | CellTracker Green probe | C2925 | Thermo Fisher Scientific Inc. (Waltham, MA, USA) |
| Davidson’s fixative | Davidson’s fixative | PH0975 | Phygene (Fuzhou, China) |
| Cyclosporine A | cyclosporine A | C106893 | Aladdin (Shanghai, China) |
| Adhesion microscope slides | microscope slides | 188105 | Citoglas (Jiangsu, China) |

**Additional file Figures.**


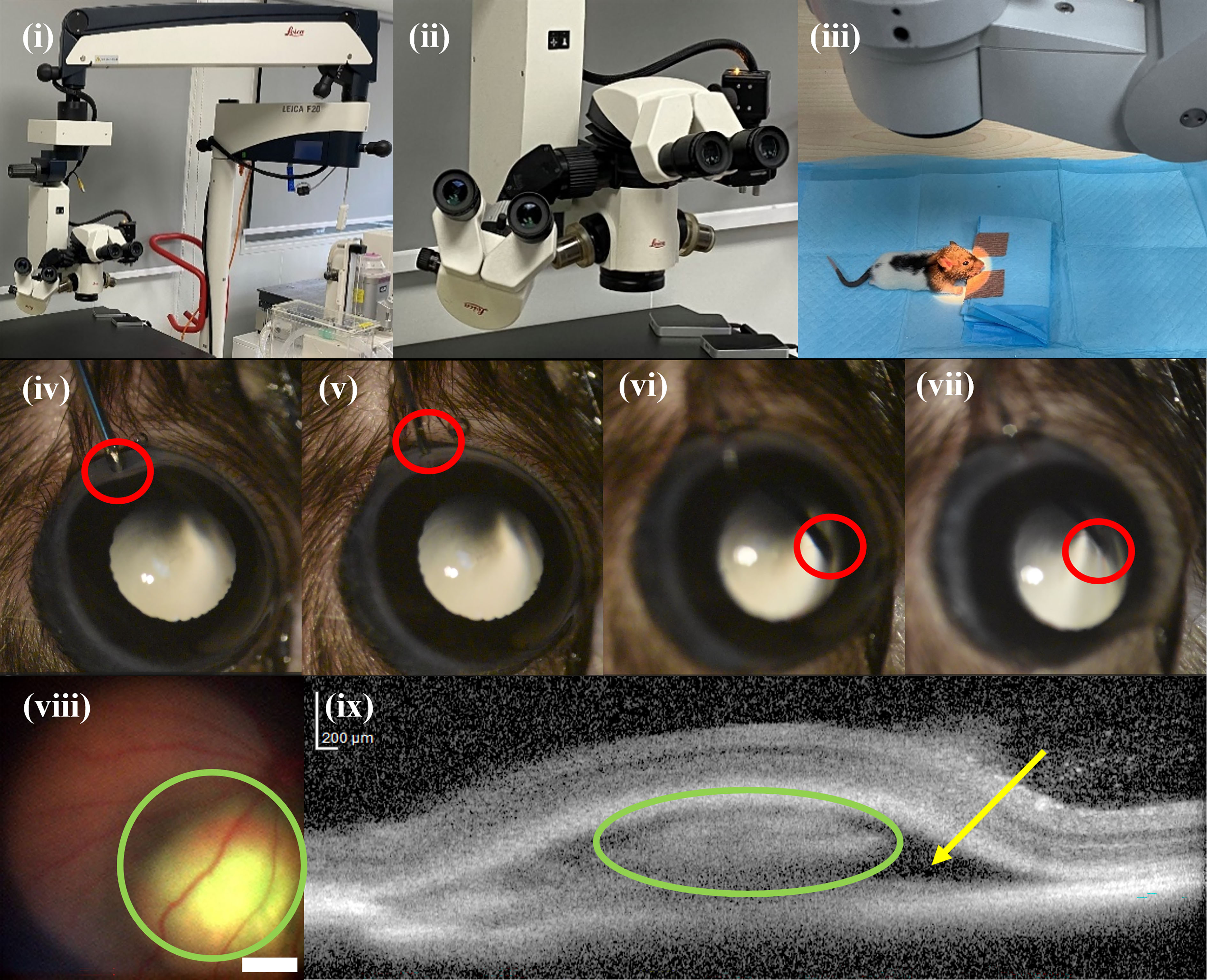


**Fig. S1.** **subretinal injection and characterization.** (ⅰ) (ⅱ) Surgical microscope used in this study. (ⅲ) Position of RCS rats under surgical microscope. (ⅳ)-(ⅶ) Detailed injection process. (ⅳ) Creating a channel at 1-2 mm behind the limbus with a 30G sharp needle. (ⅴ) A syringe with a 33G blunt needle loaded with cell was inserted through the channel. (ⅵ) The blunt needle passed through the vitreous and gently touched the retina. (ⅶ) Push the blunt needle to reach the subretinal space. (ⅷ) Fundus photograph with green fluorescence. Scale bar: 600 μm. (ⅸ) Optical coherence tomography (OCT) image of rat subjected to subretinal injection with cells. Subretinal space was pointed out by yellow arrow; cell mass was circled in green. Scale bar: 200 μm.


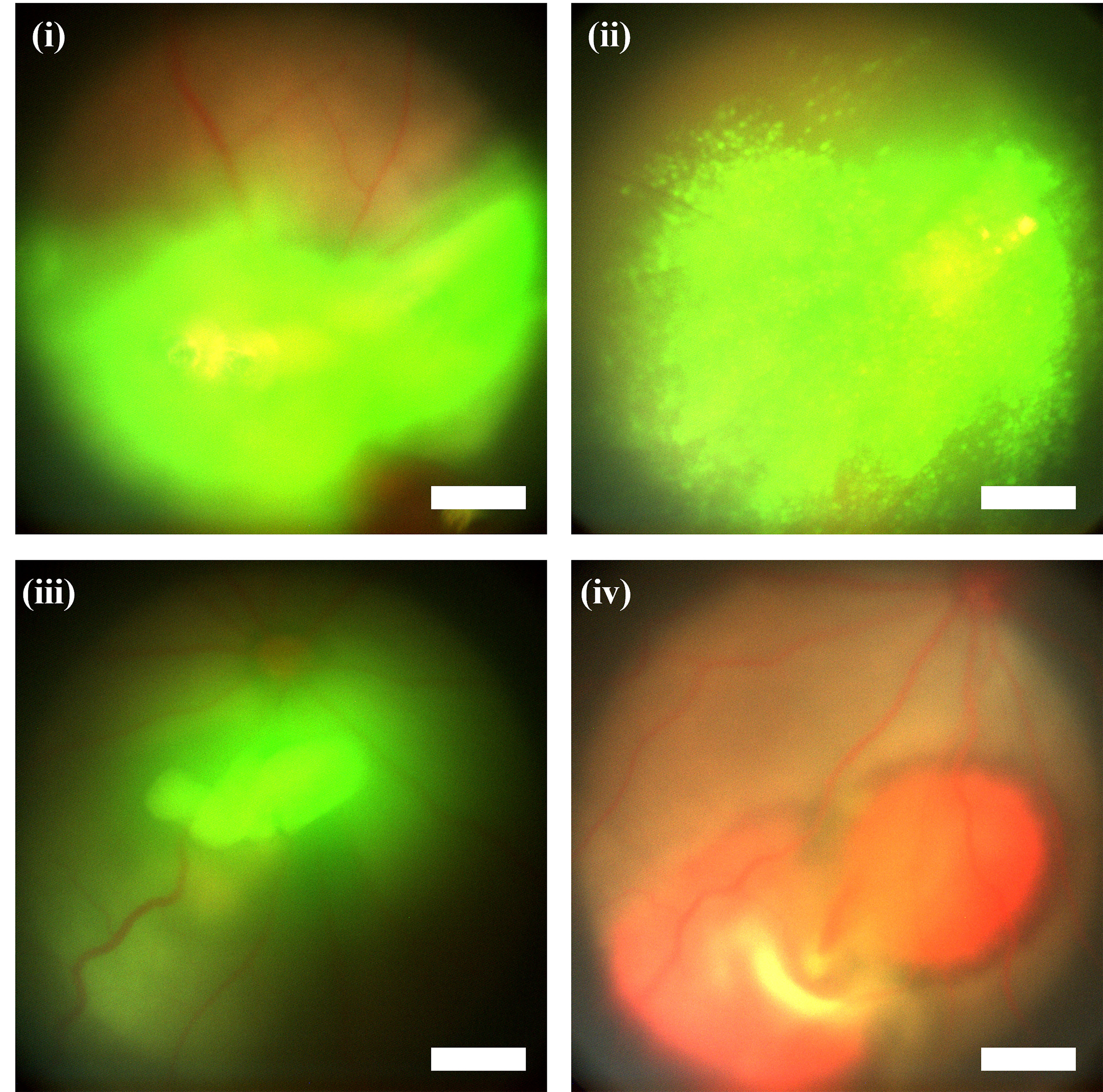


**Fig. S2.** **Exclusion criterion.** (ⅰ) (ⅱ) Cells were injected into the vitreous cavity. (ⅲ) Some cells leaked to the vitreous cavity. (ⅳ) Post-injection bleeding. Scale bar: 600 μm.


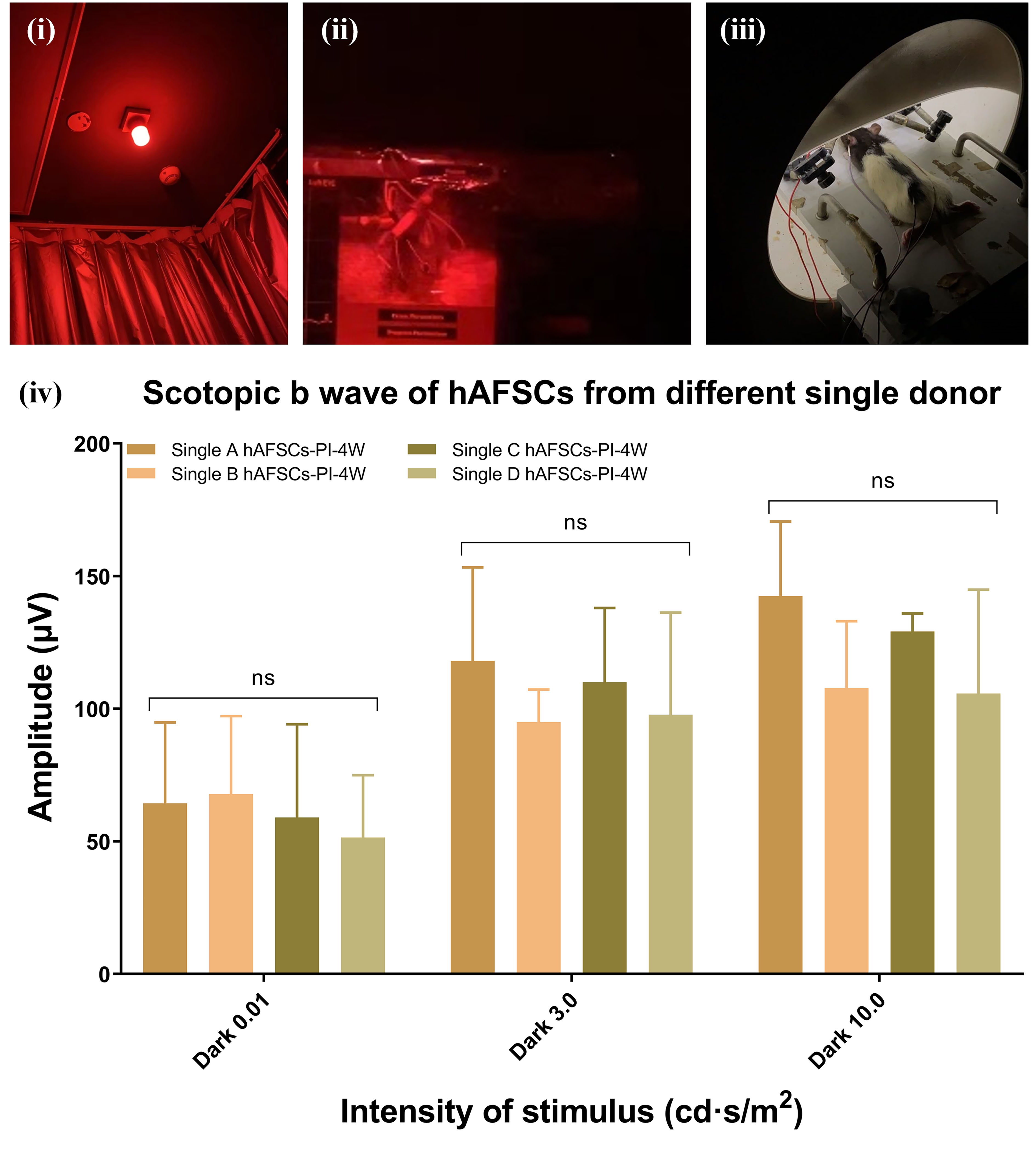


**Fig. S3. Electroretinogram performance.** (ⅰ) Dark room with dim red light during Electroretinogram (ERG) test. (ⅱ) The condition of RCS rats observed on a computer monitor during ERG test. (ⅲ) RCS rat on the platform of ERG test. (ⅳ) The amplitude of scotopic b waves of RCS rats in the dark, with adaptation at 0.01, 3.0, and 10.0 cd·s/m2 intensities, at 4 weeks post-injection; the RCS rats were subjected to subretinal transplantation of hAFSCs, which were derived from four different donors. PI: postinjection. “ns”: the difference was not significant.
